# An Approach to the Optimization of Ba-Mn-Cu Perovskites as Catalysts for CO Oxidation: The Role of Cerium

**DOI:** 10.3390/nano15191467

**Published:** 2025-09-25

**Authors:** Álvaro Díaz-Verde, María José Illán-Gómez

**Affiliations:** MCMA Group, Inorganic Chemistry Department, Materials Institute of the University of Alicante (IUMA), Faculty of Sciences, University of Alicante, 03690 Alicante, Spain; alvaro.diaz@ua.es

**Keywords:** perovskite, manganese, copper, cerium, CO oxidation

## Abstract

Two copper-containing perovskites Ba_0.8_Mn_0.7_Cu_0.3_O_3_ and Cu(4 wt%)/Ba_0.7_MnO_3_ (selected from previous studies) were tested as catalysts for the CO oxidation reaction under conditions similar to the found in the exhaust of last-generation automotive internal combustion engines. The Cu(4 wt%)/Ba_0.7_MnO_3_ sample has been selected due to its higher tolerance to CO_2_. In order to optimize the performance of this sample for the reaction under study, a Cu(2 wt%)Ce(2 wt%)/Ba_0.7_MnO_3_ formulation was synthesized, characterized and tested. The excellent catalytic performance of the bimetallic formulation, in terms of CO conversion at low temperatures and tolerance to CO_2_, is because cerium improves the redox properties and increases the proportion of reduced copper species on the surface compared to the Cu(4 wt%)/Ba_0.7_MnO_3_ sample.

## 1. Introduction

Nowadays climate change, caused by the emission of greenhouse gases (GHGs) such as carbon dioxide (CO_2_), carbon monoxide (CO), nitrogen oxides (NO_x_) and methane (CH_4_) [1,2], is considered one of the most challenging global issues. Some of these GHGs are released by the internal combustion engines (ICEs) used for mobile applications, particularly in the automotive sector [3,4,5]. Thus, to reduce the environmental impact of emissions from this sector, several emerging decarbonization technologies are intended to be applied alongside the use of electric and hybrid engines and alternative fuels, such as, among others, the optimization of the combustion process (through, for example, the implementation of the oxy-fuel combustion technology) [6,7,8]. Despite these advances, ICEs based on fossil fuels will remain predominant in the automotive sector because the alternative technologies remain being very expensive and do not offer the maturity required for a widespread use [9,10,11].

An important tool to control the emission of pollutants from ICEs exhaust is based on the use of catalysts able to promote the conversion of CO, hydrocarbons (HCs), NO_x_ and particulate matter into less harmful substances [12,13,14]. Focusing the attention on CO, which is a poisonous gas that causes high mortality rates [15,16], its removal is mainly based on the catalytic oxidation to CO_2_, being the most active catalytic formulations based on noble metals, such as Pt [17,18,19]. However, their high cost and tendency to sinter or to become poisoned are relevant drawbacks to overcome [20,21]. As an alternative, great efforts are being developed to obtain other catalytic formulations based on transition metal oxides, such as Mn, Co or Ni, that are more affordable and also catalytically efficient for the CO oxidation reaction [22,23,24].

In this context, perovskite-type oxides (ABO_3_) have been widely proposed as catalysts for the CO oxidation, as they are highly versatile solids due to their tunable properties that can be modified to promote changes in the redox properties, in the population of oxygen defects and in the oxygen mobility [25,26,27,28,29,30,31] by either adjusting the stoichiometry in the A-site (as La_0.8_MnO_3_, Sr_0.9_TiO_3_ or Sr_0.7_NbO_3_) and/or doping the B-site (such as LaCo_1-x_Cu_x_O_3_), or by being used as supports of other active phases (MnO_x_/LaMnO_3_, NiO/CaZrO_3_ and NiO/LaFeO_3_. Considering the previous research developed by the authors, the induced Ba-deficiency in the BaMnO_3_ formulation improved the catalytic performance for the CO oxidation reaction [32]. In fact, Ba_0.7_MnO_3_ perovskite presented the best performance, as the generation of oxygen vacancies and the increase of the Mn (IV) fraction allowed improving the activation of CO and O_2_ [32]. Moreover, the role of Cu as promoter of Ba-Mn perovskites has also been proven [33,34]. Considering this background, two copper-containing perovskites (Ba_0.8_Mn_0.7_Cu_0.3_O_3_ and Cu(4 wt%)/Ba_0.7_MnO_3_) selected from previous studies of the authors [33,34] were tested as catalysts for the CO oxidation reaction under conditions closer to those found in the exhaust of last-generation automotive internal combustion engines. Subsequently, in order to further optimize the best formulation (Cu(4 wt%)/Ba_0.7_MnO_3_), the Cu(2 wt%)Ce(2 wt%)/Ba_0.7_MnO_3_ sample was synthesized, characterized and tested for the CO oxidation, in which the formation of the bimetallic Cu-Ce system is expected to improve the catalytic properties of the monometallic Cu-containing sample, due to the excellent properties of CeO_2_ such as its reducibility, its oxygen mobility and its strong interaction with Cu [35,36,37,38].

## 2. Materials and Methods

### 2.1. Synthesis and Characterization

The synthesis of the Ba_0.7_MnO_3_ (B0.7M-E), and Ba_0.8_Mn_0.7_Cu_0.3_O_3_ (B0.8MC) samples was performed via the sol-gel method adapted to aqueous medium [39,40], being the Cu4/B0.7M-E and Cu12/B0.7M-E samples obtained by the incipient wetness impregnation method [41,42]. The details of these synthesis procedures can be found in previous publications of the authors [32,33,34]. Briefly, the sol-gel procedure involves the formation of a gel from an aqueous solution containing the metal precursors (as nitrates), citric acid and EDTA (although the latter one is not employed for the synthesis of B0.8MC) as gelling and chelating agents, respectively. Subsequently, the gel is dried and calcined in order to obtain the powdered samples. The synthesis of the Cu(2 wt%), Ce(2 wt%)/Ba_0.7_MnO_3_ (Cu2Ce2/B0.7M-E) sample was carried out also by the incipient wetness impregnation method of the B0.7M-E support. For that, copper (II) nitrate trihydrate (Cu(NO_3_)_2_·3 H_2_O, Panreac, 99.0 wt%) and cerium (III) nitrate hexahydrate (Ce(NO_3_)_3_·6 H_2_O, Sigma-Aldrich, St. Louis, MO, USA, 99.0 wt%) were used as precursors. During the process, the support was impregnated with the appropriate volume of solution containing both precursors simultaneously. The wet perovskite was stirred for 24 h and at room temperature, dried at 90 °C for 24 h, and finally calcined at 600 °C for 3 h.

To determine the actual copper and cerium contents, the Inductively Coupled Plasma Optical Emission Spectroscopy (ICP-OES) technique was employed, using a Perkin-Elmer device model Optimal 4300 DV. The extraction of the metals consisted of a mineralization using a diluted aqua regia solution and stirring at 60 °C for 1 h until the dissolution of the samples was achieved.

For the identification of the crystalline structure of the samples, the X-Ray Diffraction (XRD) technique was used. The XRD patterns were recorded between 20° and 80° 2θ values with a step rate of 0.4°·min^−1^ and employing a Cu K_α_ (0.15418 nm) radiation in a Bruker D8-Advance device. The average crystal size and lattice strain values of the perovskite phases were determined by the Williamson-Hall method [43,44], calculating the Y-intercept and the slope of the regression line, respectively, according to the Equation (1).

*B* cos θ = *ε*(4 sin θ) + *K λ D*^−1^ (1)

where *B* corresponds to the full width at half maximum of the XRD peaks, *ε* is the lattice strain, *K* is the shape factor (set to 0.9), *λ* is the Cu K_α_ wavelength, and *D* is the average crystal size. Furthermore, the cell parameters of the perovskite phase were calculated according to the equation for the hexagonal system (Equation (2)) and using the (110) and (101) planes.

*d_hkl_*^−2^ = (4(*h*^2^ + *hk* + *k*^2^) (3*a*^2^)^−1^) + (*l*^2^ *c*^−2^) (2)

being *d_hkl_* the interplanar spacing, *hkl* the Miller indexes, and *a* and *c* the cell parameters.

The morphology of the samples and the distribution of the chemical species was analyzed by electronic microscopy, using a ZEISS Merlin VP Compact for Field Emission Scanning Electron Microscopy (FE-SEM) combined with Energy Dispersive X-ray spectroscopy (EDX). For obtaining the FE-SEM images, a voltage of 2 kV (resolution of 1.5 nm) is applied, meanwhile, for those obtained by applying EDX, a voltage of 15 kV (resolution of 0.8 nm) is used.

The surface characterization of the samples was conducted by X-ray Photoelectron Spectroscopy (XPS), using a K-Alpha Photoelectron Spectrometer by Thermo-Scientific, with an Al K_α_ (1486.7 eV) radiation source. During the analyses, the pressure of the analysis chamber was maintained at 5·10^−10^ mbar. The binding energy and kinetic energy scales were calibrated by setting the C 1s transition at 284.6 eV, and the deconvolution of the resulting XPS profiles was performed with the Thermo Avantage software (v5.9929). During the experiments, the Ba 3d^5/2^, Mn 2p^3/2^, Mn 2p^1/2^ (data in Table S1 and Figure S1 of the Supplementary Information File) Mn 3p, O 1s, Cu 2p^3/2^, Cu L_3_M_4.5_M_4.5_ Auger signal, and Ce 3d transitions were analyzed. For the determination of the contribution of the different surface Cu species, the deconvolutions of the Cu L_3_M_4.5_M_4.5_ Auger signals for Cu, Cu_2_O and CuO references (Table S2 and Figure S2 of the Supplementary Information File), proposed by R. Peter and M. Petravic [45], have been used. After, they were adjusted according to the Auger signals registered for our samples.

The reducibility of the samples was explored with the Temperature-Programmed Reduction with H_2_ (H_2_-TPR) technique, which was carried out in a ChemBET *Pulsar* TPR/TPD device from Quantachrome Instruments (Anton Paar Austria GmbH, Graz, Austria), equipped with a Thermal Conductivity Detector (TCD). During the experiments, 30 mg of sample where heated at 10 °C·min^−1^ from 25 to 1000 °C under a 5% H_2_/Ar atmosphere (40 mL·min^−1^). The amount of H_2_ consumed was quantified by using a copper (II) oxide (CuO, Sigma-Aldrich, 99.9 wt%) reference sample, which is reduced according to Reaction (3) [46].

CuO + H_2_ → Cu + H_2_O (3)

O_2_ Temperature-Programmed Desorption (O_2_-TPD) tests were conducted in a Thermo Gravimetric Mass Spectrometer (TG-MS) system (Q-600-TA and Thermostar from Balzers Instruments (Pfeiffer Vacuum GmbH, Asslar, Germany), respectively). For the experiments, 16 mg of sample were heated at 10 °C·min^−1^ from room temperature to 950 °C under a 100 mL·min^−1^ of He. Prior to the experiments, each sample underwent a pretreatment at 150 °C for 1 h to remove the moisture. To follow the O_2_ evolved during the experiments, the 32 *m/z* signal was monitored. The quantification of the amount of oxygen evolved was performed by using a CuO reference sample, which decomposes into Cu_2_O (under in the experimental conditions used), according to Reaction (4) [47].

4 CuO → 2 Cu_2_O + O_2_ (4)

### 2.2. Activity Tests

To determine the catalytic activity for the CO oxidation, Temperature-Programmed Reaction with CO (CO-TPRe) experiments were conducted using a gas mixture composed of 1% CO and 1% O_2_ in He, as an approximation to the CO partial pressure under the actual Three-Way Catalysts (TWCs) working conditions [48]. For the experiments, 50 mg of sample and 100 mg of SiC were loaded into a U-shaped quartz reactor, which was subjected to a heating rate of 10 °C·min^−1^ until 500 °C under a 100 mL·min^−1^ flow (Gas Hourly Space Velocity (GHSV) of 4967 h^−1^) of the gas mixture. Prior to the CO-TPRe tests, the sample-SiC mixture was pretreated for 1 h at 600 °C under a 5% O_2_/He gas mixture to clean the surface of the samples. The commercial 1% Pt/Al_2_O_3_ sample (Sigma-Aldrich), used as a reference, was not subjected to the pretreatment in order to minimize the Pt sintering [49]. On the other hand, some selected samples were subjected to stability tests consisting of one or two reaction cycles at 250 and 300 °C (for 5 h). Finally, to test the selected samples under conditions even closer to that of TWCs, CO_2_ (15%) was included in the reactant mixture and long-term cycles of 5 h and 20 h were performed. Before each cycle, the sample was subjected to the preheating treatment previously described. For the determination of the gaseous stream composition, an Agilent 8860 Gas Chromatograph, equipped with a TCD and two packed columns (Porapak-Q and MolSieve-13X from Agilent Technologies Spain, Madrid, Spain), was used. The percentage of CO conversion (C_CO_) was calculated by the Equation (5):

*C_CO_* (%) = (*CO_in_* − *CO_out_*) (*CO_in_*)^−1^ · 100 (5)

being *CO_in_* and *CO_out_* the inlet and outlet molar flow rates, respectively. Additionally, the change of the CO conversion (Δ*C_CO_*) and the CO specific activity (*a_CO_*) values during the stability tests were calculated by using the Equations (6) and (7):

*ΔC_CO_* (%) = *C_CO,f_* − *C_CO,i_* (6)

*a_CO_* = (*CO_in_* − *CO_out_*) (*n_Cu_*)^−1^ (7)

where *C_CO,f_* and *C_CO,i_* are the final and initial CO conversions during the isothermal test, respectively; and *n_Cu_* the number of moles of Cu in the sample. The CO signals recorded by the gas chromatograph were calibrated by using a calibration gas mixture composed by 5083 ± 102 ppm Ar, 5.491 ± 0.055% CO in He (expanded error with a coverage factor (k) of 2).

## 3. Results and Discussion

### 3.1. Selection of the Perovskites

Previous studies focused on the catalyzed CO oxidation reaction developed by the authors [33,34] revealed that:

* From a Cu/Ba_0.7_MnO_3_ series of samples, with nominal Cu contents of 4, 8 and 12 wt% (denoted as Cux/B0.7M-E, x = 4, 8 and 12), the Cu4/B0.7M-E and Cu12/B0.7M-E formulations feature the best catalytic performance as they present a high proportion of Cu species with a strong interaction with the perovskite support [34].
* From the Ba_x_Mn_0.7_Cu_0.3_O_3_ series of samples (with x = 1, 0.9, 0.8 and 0.7), the Ba_0.8_Mn_0.7_Cu_0.3_O_3_ composition (denoted as B0.8MC) shows the best catalytic behavior due to its high amount of Mn (IV) ions, oxygen vacancies and reduced copper species (Cu (I)), that are more active than oxidized Cu species (Cu (II)) [33].

**Figure 1:**
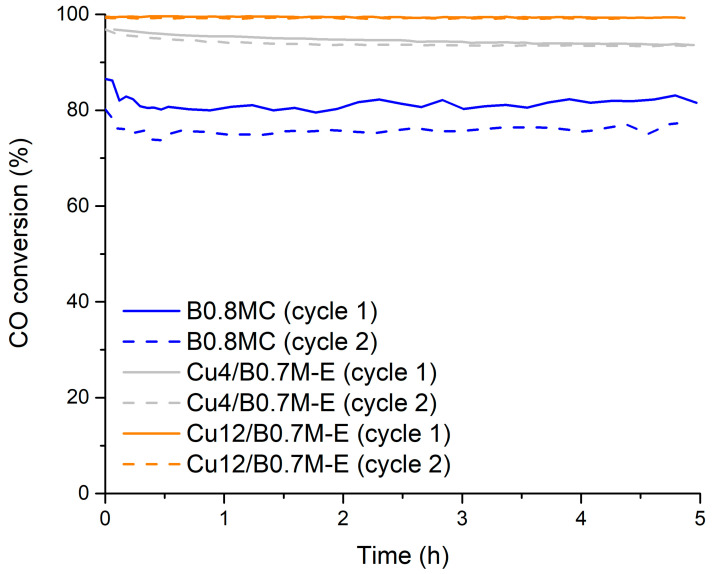
CO conversion profiles of the B0.8MC, Cu4/B0.7M-E and Cu12/B0.7M-E samples at 300 °C, under a 1% CO, 1% O_2_ in He reactant mixture.

Considering these findings, in this paper we compare the B0.8MC perovskite with Cu4/B0.7M-E and Cu12/B0.7M-E samples, in order to determine a potential effect of the Cu loading method on the catalytic performance for the CO oxidation reaction. The most relevant characterization and activity data for these three samples are summarized in Table 1 and Figure 1.

Both Cu-impregnated samples exhibit a higher CO specific activity than B0.8MC under the conditions tested, suggesting that the impregnation method is the most effective for achieving a high activity and stability. According to the characterization data, the two samples obtained by impregnation present a higher fraction of Mn (IV) on the surface than the B0.8MC perovskite [50], showing Cu12/B0.7M-E also a higher amount of surface copper species, as indicates the Cu/(Ba+Mn+Cu) ratio (which provides information about the distribution of the Cu species on the samples [51]). This is because the XPS ratio is higher than the nominal one for the Cu12/B0.7M-E sample, meanwhile the opposite is found for the B0.8MC perovskite. Additionally, in order to select one of the two perovskites obtained by impregantion, it was considered that the fraction of copper with a strong interaction with the perovskite is a key factor for achieving a high CO conversion [32,33,34]. Thus, as the Cu_si_/Cu_wi_ ratio (being Cu_si_ and Cu_wi_ the contributions of the Cu species with strong and weak interactions with the support, respectively) is higher for the Cu4/B0.7M-E sample than for Cu12/B0.7M-E and, as both samples feature a similar reducibility (see the H_2_-TPR data in Table 1), Cu4/B0.7M-E has been finally selected to be compared with B0.8MC for the CO oxidation reaction under more demanding conditions than those used in the previous publications [33,34]. To achieve this purpose, the following CO oxidation tests have been developed:

(i) for testing the stability of the samples at a temperature lower than 300 °C, two reaction cycles at 250 °C under the 1% CO, 1% O_2_ in He reactant mixture.
(ii) in order to determine the tolerance of the catalysts to CO_2_ [25], a CO oxidation reaction at 300 °C using the 1% CO, 1% O_2_ in He reactant mixture, in which a 15% of CO_2_ was added to simulate the average composition typically found in the actual gasoline exhaust.

### 3.2. Effect of the Reaction Temperature for the CO Oxidation

**Figure 2:**
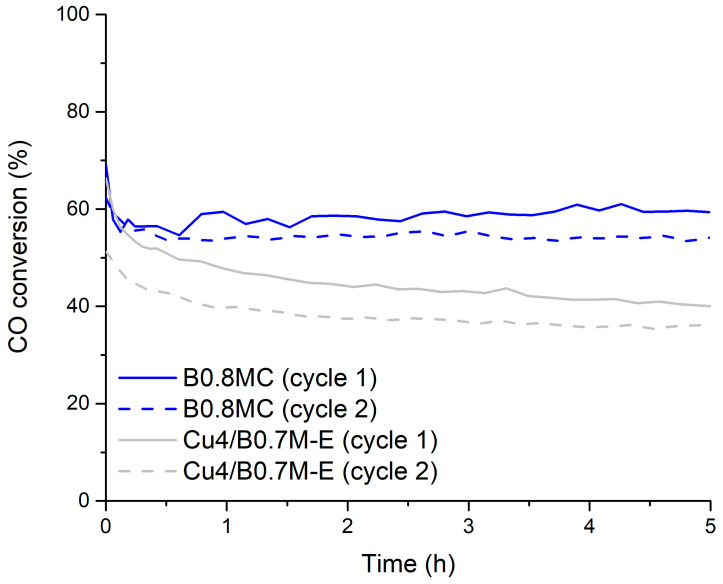
CO conversion profiles of the B0.8MC and Cu4/B0.7M-E samples at 250 °C and under a 1% CO, 1% O_2_ in He reactant mixture.

Figure 2 shows the CO conversion profiles at 250 °C, under the 1% CO, 1% O_2_ in He reactant mixture, being the related data displayed in Table 2. During the two reaction cycles at 250 °C, B0.8MC features higher CO conversions than Cu4/B0.7M-E, but the CO specific activity is lower for the former due its higher Cu percentage (8.845 wt% versus 3.946 wt% for B0.8MC and Cu4/B0.7M-E, respectively). Note that, for both samples, the CO conversion is slightly lower in the second cycle and, even though both samples present a good stability versus time, Cu4/B0.7M-E exhibits a slightly more pronounced deactivation with time than B0.8MC.

The CO conversion at 250 °C of B0.8MC is lower than the published by Y. Yang [52] for a LaCo_0.9_Ni_0.1_O_3_ perovskite tested at 270 °C under a 1% CO, 2% O_2_ in He reactant atmosphere (approximately 90% after 5 h of reaction), which should be consequence of the higher reaction temperature (270 °C versus 250 °C) and of the excess of O_2_ (2% versus 1%). However, the catalytic performance of B0.8MC is better than the registered for a series of LaNiO_3_ perovskites calcined at different temperatures (600–800 °C) at 200 °C and under a similar reactant atmosphere [53], probably due to the higher reaction temperature used (250 °C versus 200 °C) 

In order to understand the trend in the catalytic performances shown by the two samples, a deep characterization of the samples used in the two reaction cycles (denoted as used samples) has been carried out.

The XPS data of the fresh and used samples are collected in Table 3 for a direct comparison. The lower values of the Mn(IV)/Mn(III) and of the O_L_/(Ba+Mn(+Cu)) ratios for both used samples indicate that, during the reaction at 250 °C, a decrease in the proportion of surface Mn (IV) and an increase in the amount of surface oxygen defects/vacancies takes place. The Cu/(Ba+Mn+Cu) ratios of the fresh and used samples are similar, suggesting that the distribution of the copper species is not significantly modified during the reaction. Finally, the lower value of the BaCO_3_/Ba_L_ ratio (which informs about the presence of carbonate groups on the surface) reveals that the Cu4/B0.7M-E sample presents a lower degree of carbonation after the reaction. Thus, as for both samples the amount of surface oxygen vacancies (which act as active sites for oxygen activation) increases, but the Mn (IV) surface proportion decreases (more significantly for Cu4/B0.7M-E than for B0.8MC), it seems that the latter factor is more relevant for determining the catalytic performance than the former.

**Figure 3:**
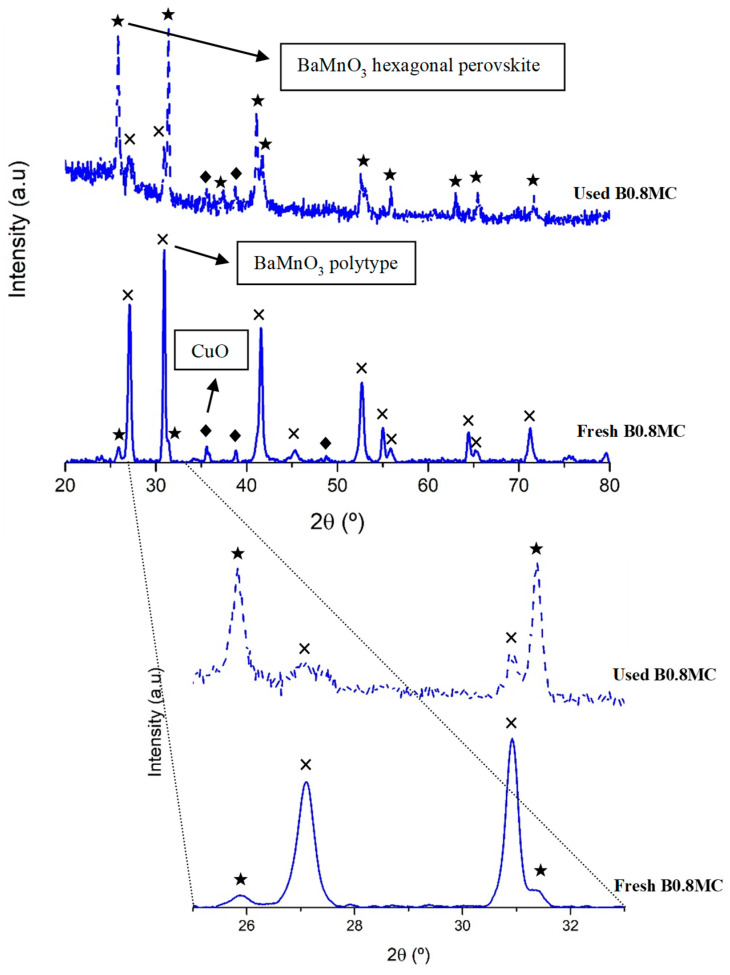
XRD patterns of the fresh and B0.8MC sample used in the CO oxidation reaction at 250 °C.

To deeper explore the changes of the surface Cu species during the reaction at 250 °C, the Cu L_3_M_4.5_M_4.5_ Auger signal of the used samples has been analyzed [54,55]. As the data in Table 3 reveal, the higher stability shown by the B0.8MC sample seems being due to the presence of Cu (I) species along the reaction time [56] as the Auger signal appears at the same position for the fresh and used samples. Note that, for the B0.8MC sample, Cu (I) and Cu (II) species coexist on the surface since the characteristic satellite peaks of Cu (II) in the Cu 2p^3/2^ spectrum are detected [57,58]. Additionally, by comparing the XRD patterns of the fresh and used B0.8MC formulation (Figure 3), it seems that an exsolution of the Cu species to the surface is taking place during the reaction, as a transition of the main crystalline phase from the polytype perovskite (which is a distortion of the corresponding hexagonal perovskite promoted by the insertion of Cu into the perovskite network [59]) to the BaMnO_3_ hexagonal perovskite structure is detected. This result aligns with the change in the percentages of the surface Cu species, estimated by using the Cu L_3_M_4.5_M_4.5_ Auger signal (Table 3), as a decrease of the proportion of Cu species with a strong interaction with the perovskite (as consequence of the observed exsolution) is detected. On the other hand, for the fresh Cu4/B0.7M-E formulation, the Auger signal could be assigned both to Cu (0) or Cu (II) [56], being the latter one the most probable considering the sample background [34]. However, for the used Cu4/B0.7M-E sample, the Auger signal is shifted suggesting the presence of Cu (I), which is confirmed by the evolution of the percentages obtained with the Auger signal. So, as Cu (I) is more effective than Cu (II) for the CO activation [60,61], a gradual increase of the CO conversion would be expected for the Cu4/B0.7M-E sample. However, the CO conversion follows just the opposite trend. Note that, a decrease of the CO conversion would be expected if a higher proportion of Cu is present on the surface of the impregnated sample, as larger Cu particles (so, more agglomerated) should be formed. However, the Cu/(Ba+Mn+Cu) ratio of the used Cu4/B0.7M-E does not support this hypothesis.

In summary, the decrease of the proportion of Mn (IV) on the surface of the Cu4/B0.7M-E sample seems to cause the slight loss of CO conversion along the reaction time. Meanwhile, the presence of Cu (I) on the B0.8MC fresh and used samples seems justifying its better catalytic performance.

### 3.3. Tolerance to CO_2_

**Figure 4:**
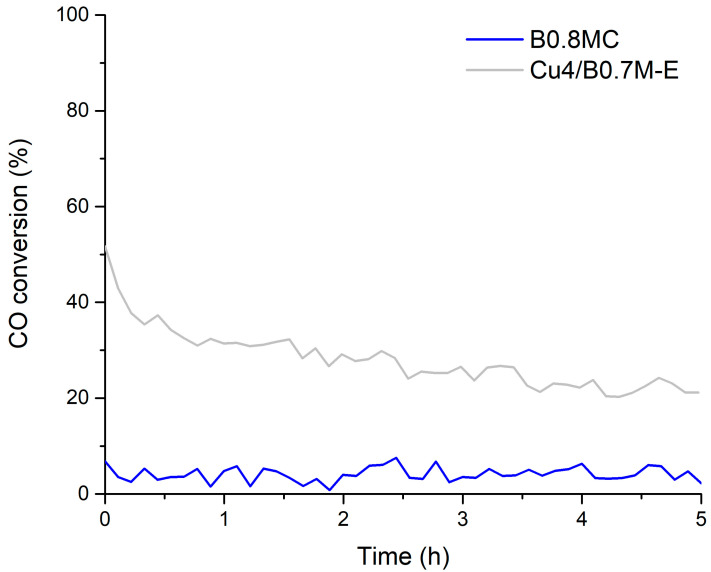
CO conversion profiles of the B0.8MC and Cu4/B0.7M-E samples at 300 °C (under a 1% CO, 1% O_2_ in He reactant atmosphere) in the presence of 15% CO_2_.

Figure 4 and Table 4 display the data obtained for the CO oxidation at 300 °C in the presence of CO_2_. Both samples undergo a clear deactivation as the CO conversions and the CO specific activities are significantly lower than in the absence of CO_2_ (see Figure 1 and Table 1). Similar results are published in the literature for perovskite and spinel-type samples, being the inhibition of CO_2_ assigned to the competition of CO and CO_2_ by the same adsorption sites, and to the formation of carbonate groups after CO_2_ adsorption [62,63,64]. However, the tolerance to CO_2_ of the Cu4/B0.7M-E sample is higher than that of the B0.8MC one, which becomes to be completely inactive at the end of the reaction time.

**Figure 5:**
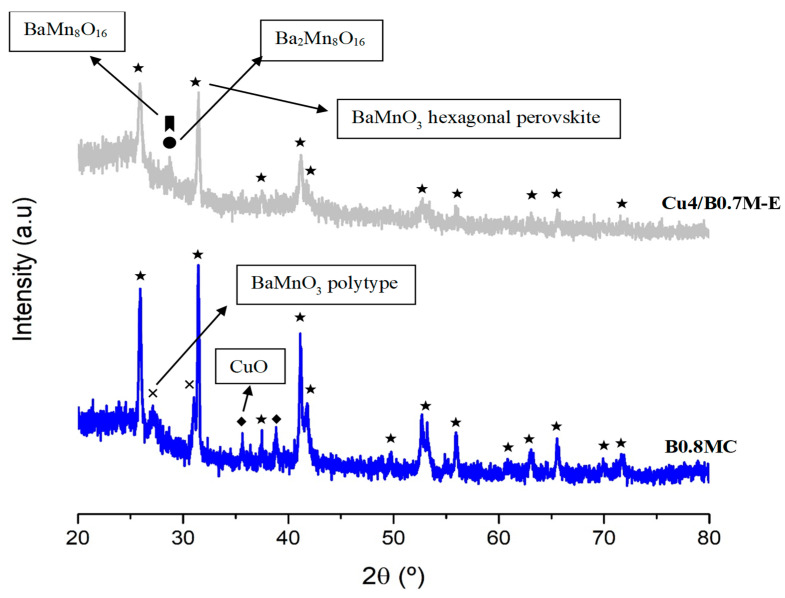
XRD patterns of the used B0.8MC and Cu4/B0.7M-E samples in the stability tests performed at 300 °C in the presence of 15% CO_2_.

The used samples have been characterized by XRD and XPS to determine the potential structural and/or surface modifications occurred during the reaction, being the results shown in Table 5 and Figure 5.

The analysis of the Ba 3d^5/2^ transition reveals an increase in the amount of carbonate groups on the surface of the used Cu4/B0.7M-E sample. However, as this BaCO_3_ phase is not detected by XRD (Figure 5), it should be amorphous or, if it is crystalline, its amount should be below the detection limit of the XRD technique. Moreover, the XPS O_L_/(Ba+Mn(+Cu)) ratio for the used samples is lower than for the fresh formulations, showing the Cu4/B0.7M-E sample the most pronounced decrease. This fact suggests the formation of oxygen vacancies on the surface of the two perovskites during the reaction (as it was also detected after testing at 250 °C), which takes place along with a decrease of the amount of surface Mn (IV) species, as deduced from the change in the Mn(IV)/Mn(III) ratio. Note that these two modifications are more relevant for the impregnated sample. Additionally, the decrease of the Cu/(Ba+Mn+Cu) ratios for the two used samples suggests that copper species seem being partially inserted into the inner structure of the perovskite during reaction. However, the XRD profile of the used B0.8MC sample shows a transition from the BaMnO_3_ polytype structure to the hexagonal structure suggesting just the opposite, that is, the exsolution of the Cu species (since it is the Cu insertion which causes the formation of the polytype structure). On the contrary, regarding the used Cu4/B0.7M-E formulation, the support still presents the hexagonal perovskite structure, like in the fresh sample [32]. Therefore, considering the decrease of the Cu/(Ba+Mn+Cu) ratio observed for the two used samples, it seems that the higher amount of oxygen vacancies generated during the reaction on both samples avoids the distortion of the perovskite structure due to the insertion of copper into the lattice [65]. Indeed, as observed after reaction at 250 °C, the Auger Cu L_3_M_4.5_M_4.5_ signals (see Table 5) reveal the preservation of the copper species with a strong interaction with the perovskite (including also those with an oxidation state a lower than Cu (II)) during the reaction on the surface of the B0.8MC and Cu4/B0.7M-E samples, which is confirmed by the percentages of the Cu_si_ species on the surface. Consequently, it seems that, for B0.8MC, the partial insertion of the copper species into the bulk during the reaction hindered their interaction with the reactants, causing the decrease of the CO conversion in the presence of CO_2_. For the Cu4/B0.7M-E sample, the increase of the proportion of carbonate groups during the reaction should cause the gradual deactivation featured in Figure 4.

Summarizing, the characterization of the used samples allows concluding that the decrease of the proportion of surface Mn (IV) species, the insertion of the copper species into the bulk for both samples, and the increase of the amount of surface carbonate groups, seem causing the worse catalytic performance in the presence of CO_2_. Thus, as the tolerance to CO_2_ featured by Cu4/B0.7M-E sample is better than the shown by B0.8MC, the former formulation has been selected to be optimized in order to achieve a higher CO conversion and an improved tolerance to CO_2_.

### 3.4. Optimization of the Cu4/B0.7M-E Formulation

Based on the redox equilibrium shown in Reaction 8 and, to try to increase the fraction of Cu (I) species on the surface of the Cu4/B0.7M-E sample, the catalytic activity for the CO oxidation reaction of a series of Cu-Ce bimetallic samples supported on the B0.7M-E perovskite were explored during the development of an academic research work supervised by the authors.

Cu^2+^ + Ce^3+^ ⇆ Cu^+^ + Ce^4+^ (8)

Thus, a series of bimetallic samples with different Cu and Ce contents (4-0, 3-1, 2-2, 1-3 and 0-4, as wt% Cu–wt% Ce) were synthetized, characterized and tested under the 1% CO, 1% O_2_ in He reactant atmosphere, showing the 2-2 sample the best results as it overcomes the catalytic performance featured by the Cu4/B0.7M-E formulation under temperature-programmed CO oxidation reaction conditions. Considering this conclusion, the 2-2 sample (denoted as Cu2Ce2/B0.7M-E) was prepared, characterized and tested for the CO oxidation reaction under the most realistic conditions used in this paper.

#### 3.4.1. Chemical, Morphological and Structural Characterization

The actual Ce and Cu weight percentages of the Cu2Ce2/B0.7M-E formulation have been determined by ICP-OES, being 0.948 ± 0.073 wt% for Ce and 1.796 ± 0.003 wt%, for Cu.

**Figure 6:**
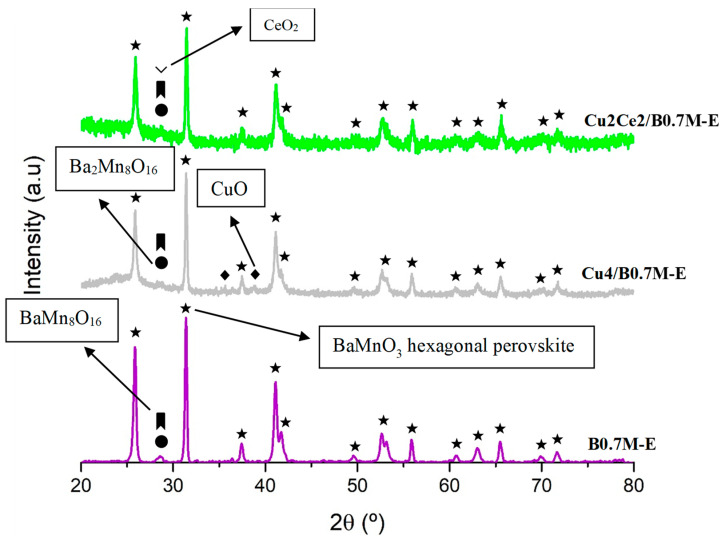
XRD patterns of the fresh B0.7M-E, Cu4/B0.7M-E and Cu2Ce2/B0.7M-E samples.

Figure 6 and Table 6 present the XRD data of the Cu2Ce2/B0.7M-E formulation, as well as the data of the support (B0.7M-E) and of the monometallic sample (Cu4/B0.7M-E) for comparative purposes. As expected, relevant structural modifications respect to the B0.7M-E support are not detected after the impregnation with Cu and Ce. Note that the CuO peaks (JCPDS-ICDD 80-0076) are not clearly distinguishable in the XRD pattern of the bimetallic sample due to the low percentage of Cu [34]. For the bimetallic sample, the main peak of CeO_2_ (JCPDS-ICDD 34-0394) overlaps with those corresponding to the BaMn_8_O_16_ (JCPDS-ICDD 29-188) and Ba_2_Mn_8_O_16_ (JCPDS-ICDD 78-962) minority phases of the support. The cell parameters included in Table 6 confirm that the incorporation of both metals does not significantly distort the perovskite structure [66], but an increase of the lattice strain values is found. According to the literature, the increase of the lattice strain in supported samples can be caused by the formation of oxygen vacancies on the support, and by the presence of an interface that separates two crystalline structures with different atomic coordination [67,68]. Moreover, the addition of Cu and Ce results in an increase of the perovskite crystal size, which was also referred by other authors for cerium-containing oxides [69].

**Figure 7:**
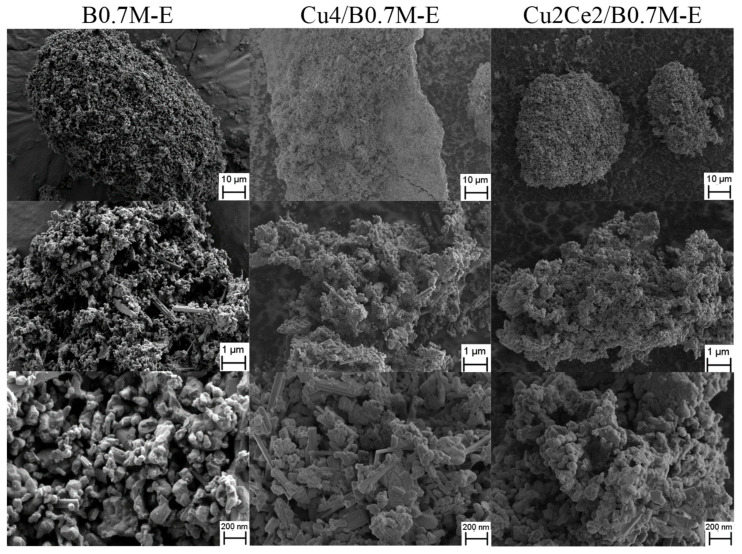
FE-SEM images of B0.7M-E, Cu4/B0.7M-E and Cu2Ce2/B0.7M-E samples.

Regarding the morphological characterization of the samples, Figure 7 shows a selection of FE-SEM images at different magnifications for the three samples. As observed, significant morphological differences are not observed between the raw B0.7M-E support and the impregnated samples, since for all of them amorphous particles with different sizes are identified. Furthermore, FE-SEM-EDX data (see Figures S3 and S4 in the Supplementary Information file) give information about the Ba, Mn, Cu and Ce distribution in the Cu4/B0.7M-E and Cu2Ce2/B0.7M-E samples. Focusing the attention on the Cu and Ce species, the CuO phase seems to be well distributed on the B0.7M-E support, showing few aggregates in certain regions of the surface, meanwhile, the Ce species seem to be very well dispersed on the surface of Cu2Ce2/B0.7M-E.

#### 3.4.2. Surface Properties

**Figure 8:**
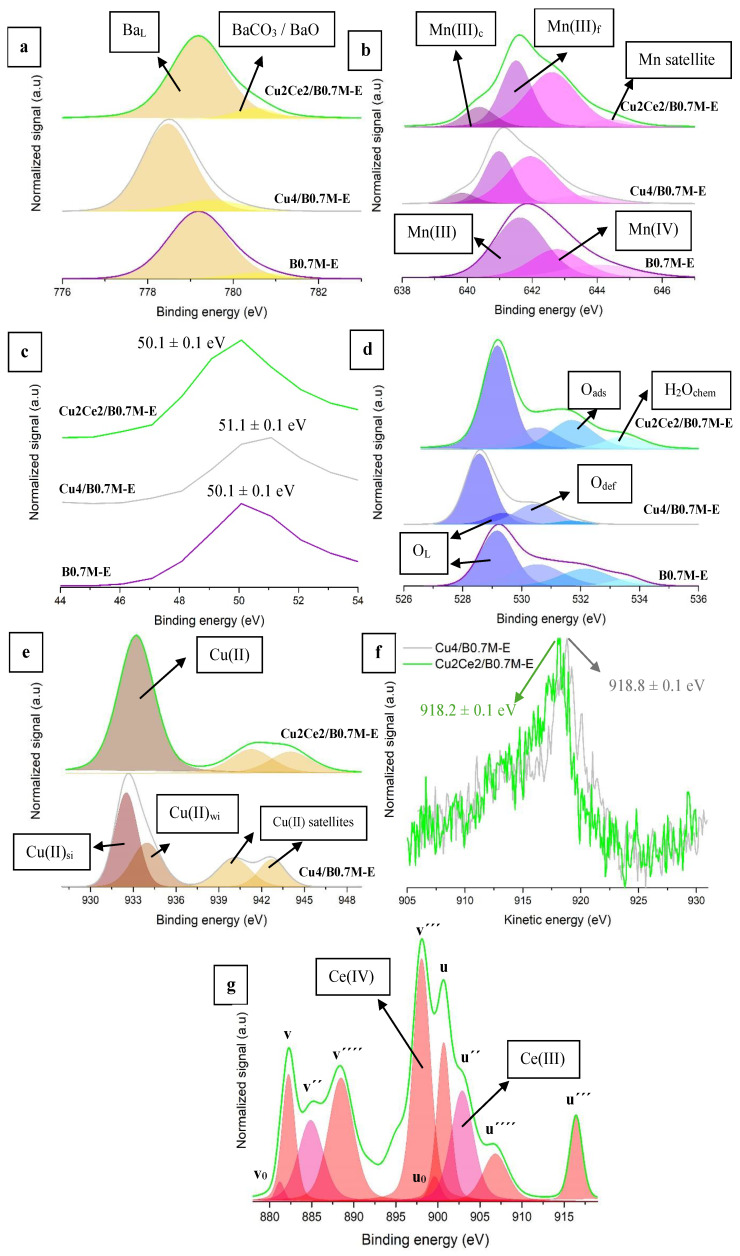
XPS spectra of the Ba 3d^5/2^ (**a**), Mn 2p^3/2^ (**b**), Mn 3p (**c**), O 1s (**d**), Cu 2p^3/2^ (**e**), Cu L_3_M_4.5_M_4.5_ Auger signal (**f**) and Ce 3d (**g**) transitions.

Figure 8 and Table 7 feature the main XPS data of the bimetallic and monometallic formulations and of the perovskite used as support.

The Ba 3d^5/2^ spectra (Figure 8a) show the two expected contributions for the Ba-based perovskites [70,71]: (i) lattice Ba (Ba_L_) at lower binding energies, and (ii) barium carbonate (formed due to the air exposition of the samples [72,73]) and barium oxide at higher binding energies. In the spectra, a chemical shift of the Ba 3d^5/2^ signal towards lower binding energies is observed, as the presence of Cu species on the surface increases the electronic density of Ba [74]. Note that, after the incorporation of Ce, the position of the Ba 3d^5/2^ signal comes back to the initial binding energy determined for the support. This fact suggests that the interaction between Cu and the perovskite support is weakened, probably as a result of the stronger interaction between Cu and Ce species. J. Jiang et al. proposed that the strong interaction between Cu and Ce is due to the formation of a solid solution [75], which probably exists on the surface of the Cu2Ce2/B0.7M-E sample. Finally, the degree of carbonation of the Ce-containing sample, as deduced from the BaCO_3_/Ba_L_ ratio, is similar to the detected for the raw support, so, Cu4/B0.7M-E seems being the sample most susceptible to be carbonated.

Regarding the Mn 2p^3/2^ signal (Figure 8b), the presence of Mn (III) (at lower binding energies) and Mn (IV) (at higher binding energies) is observed for the bimetallic sample [76,77], in which a satellite peak between 644 and 645 eV is also found [78,79]. According to the XPS Mn(IV)/Mn(III) ratio (Table 7), the fraction of Mn (IV) decreases after the addition of Ce. In fact, the Mn 3p signal (used to determine the predominant oxidation state of Mn [80], and displayed in Figure 8c) appears at the same binding energy than for the B0.7M-E, confirming the presence of a lower fraction of Mn (IV). Additionally, for both impregnated samples, a splitting of the Mn (III) signal in two deconvolutions is observed, that corresponds to Mn (III) ions close to the Cu and Ce species (Mn (III)_c_) and those far from them (Mn (III)_f_). The values of the Mn(III)_c_/Mn(III)_f_ ratio in Table 7 reveal a higher proportion of the Mn (III)_c_ species on the bimetallic sample respect to the monometallic one, which should be due to the interaction of Ce with the Mn species.

The O 1s spectra (Figure 8d) displays the four contributions expected for perovskite-type oxides [81,82]: (i) lattice oxygen (O_L_) around 529 eV, (ii) oxygen species with low oxygen coordination, that correspond to oxygen vacancies formed on the surface (O_def_), at approximately 531 eV, (iii) adsorbed oxygen, hydroxyl and carbonate groups (O_ads_) on the surface at around 532 eV, and (iv) chemisorbed water (H_2_O_chem_) at approximately 533 eV. As observed in previous studies [34], the additional thermal step included in the impregnation method removes the chemisorbed water molecules from the surface of the Cu4/B0.7M-E, but not for the Cu2Ce2/B0.7M-E sample [83]. As in previous sections, the O_L_/(Ba+Mn) ratios have been calculated to obtain information about the presence of oxygen defects. Thus, as the XPS O_L_/(Ba+Mn) ratios are lower than the nominal one, the samples present oxygen defects on the surface, being the proportion much higher for the bimetallic formulation as they are generated to balance the positive and negative charges on the surface after the Ce addition. In fact, H. Gao and colleagues proposed that the interaction between the Ce species and the MnO_6_ octahedra leads to coulombic repulsions that provoke the Jahn-Teller effect and favoring the formation of oxygen defects [84,85].

The analysis of the Cu 2p^3/2^ (Figure 8e) and of the Auger Cu L_3_M_4.5_M_4.5_ signals (Figure 8f) allows the identification of the copper species present on the surface. Thus, the band at around 933 eV, and of the satellites at higher binding energies in the Cu 2p^3/2^ profiles, reveals that Cu (II) species exist on the surface [86,87]. Additionally, the deconvolution of the main Cu 2p^3/2^ band of the Cu4/B0.7M-E sample indicates the presence of two Cu (II) species with different interactions with the support, that are those with a strong interaction with the support (Cu (II)_si_), and the others with a weak interaction with it (Cu (II)_wi_). However, for the Cu2Ce2/B0.7M-E sample, the Cu 2p^3/2^ band presents a symmetric shape, so, the two Cu (II) species with different interactions with the support do not seem to be present. This finding is consistent with the weaker interaction of the supported phases with the perovskite proposed to justify the binding energy change of the Ba 3d^5/2^ signal. Furthermore, the Auger Cu L_3_M_4.5_M_4.5_ signal of the bimetallic sample appears at 918.2 eV, likely due to the presence of reduced copper species, being this fact confirmed by the lower percentage of Cu (II) for the bimetallic sample [56,88].

The Ce 3d transition shown in Figure 8g includes the Ce 3d^5/2^ and Ce 3d^3/2^ contributions, being the former extended from 878 to 898 eV approximately, and the latter located between 898 and 918 eV [89,90]. In the Ce 3d^5/2^ contribution, the *v* and *v*‴ deconvolutions correspond to Ce (IV), the *v*_0_ and *v*″ to Ce (III), and *v*⁗ to a satellite peak that confirms the presence of Ce (IV). On the other hand, Ce 3d^3/2^ presents *u* and *u*‴ as deconvolutions of Ce (IV), *u_0_* and *u*″ due to the presence of Ce (III), and *u*⁗ corresponding to the Ce (IV) satellite [89,90]. The Ce(IV)/Ce(III) ratio (Table 7) reveals that Ce (IV) species are predominant on the surface, which supports the increase in the amount of reduced copper species (Cu(0) and/or Cu(I)) formed by the redox reaction between Cu (II) and Ce (III) species (Reaction (8)).

Finally, the nominal and experimental Cu/(Ba+Mn+Cu+Ce) and Ce/(Ba+Mn+Cu+Ce) ratios of the bimetallic sample suggest an accumulation of both metallic species on the surface. 

#### 3.4.3. Redox Properties

**Figure 9:**
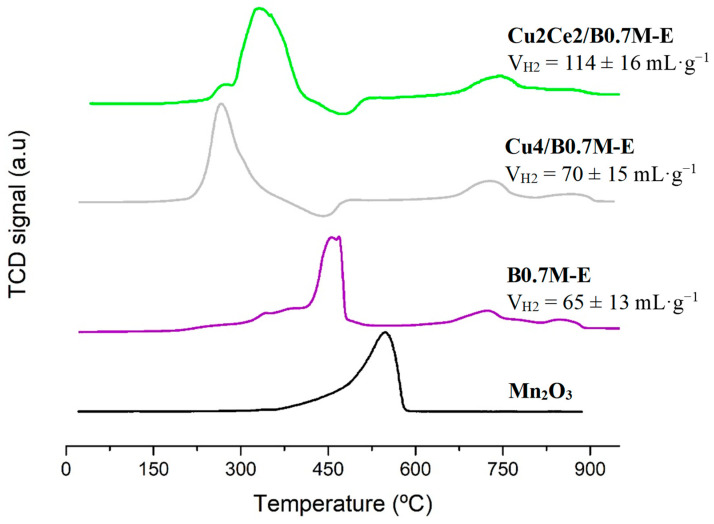
H_2_-TPR consumption profiles of the B0.7M-E, Cu4/B0.7M-E and Cu2Ce2/B0.7M-E samples, including Mn_2_O_3_ as reference. The total amount of H_2_ consumed has been included.

In the H_2_-TPR profiles of the Cu2Ce2/B0.7M-E, B0.7M-E and Cu4/B0.7M-E samples, featured in Figure 9, the peaks expected for Mn-containing perovskites [91,92] are detected: (i) from Mn (IV) and Mn (III) to Mn (II) between 400 and 500 °C, (ii) those involving oxygen species, between 700 and 800 °C, and (iii) from bulk Mn (III) to Mn (II) at around 900 °C. Additionally, at a lower temperature than that corresponding to the maximum of the main reduction peak, a shoulder due to the reduction of Mn (IV) to Mn (III) is found. On the other hand, the peak assigned to the reduction of the CuO, between 200 and 400 °C [93,94], should be present, as well as the signals due to the Ce reduction [95,96] (at 570 °C approximately for the surface Ce (IV) species, and around 750 °C for the bulk Ce (IV)). In the profiles of both impregnated samples, the maximum of the most intense reduction peak is shifted towards lower temperatures compared to the B0.7M-E perovskite, because it includes the reduction of the Cu (II) species. Focusing now on Cu2Ce2/B0.7M-E sample, it is evident that the reduction of the Mn species is facilitated by the presence of copper and cerium due to the Cu-Mn-Ce synergistic effect [97,98], being the reduction of the three metals included in the main reduction peak. However, the shift of this main reduction peak for the bimetallic sample is not as significant as in Cu4/B0.7M-E, probably due to the weaker interaction between the metallic phases and the perovskite support, above suggested. Finally, note that, for all the samples, the main reduction signals are shifted towards lower temperatures respect to the Mn_2_O_3_ used as reference (included in Figure 8). This shift should be mainly due to the different environment of the Mn ions in the perovskites, that includes also the presence of oxygen vacancies [99,100].

On the other hand, the Cu2Ce2/B0.7M-E sample presents the highest H_2_ consumption, surpassing even the theorical value [32,34] calculated assuming that only Mn (IV), Cu (II) and Ce (IV) are present (74 mL·g^−1^), or that only Mn (III), Cu (II) and Ce (IV) exist in the formulation (39 mL·g^−1^). This result reveals that the assumptions used in the theorical calculations should be incorrect for Cu2Ce2/B0.7M-E. Thus, a high H_2_ consumption could be observed if a H_2_ spillover phenomenon would take place. In fact, the spillover phenomenon, which involves the dissociative adsorption of the H_2_ molecules and the storage of the resulting H atoms as hydroxyl groups [101,102], has been proposed in the literature for copper-cerium-based formulations. Consequently, it seems that the high H_2_ consumption detected for the bimetallic sample is related to a spillover phenomenon.

**Figure 10:**
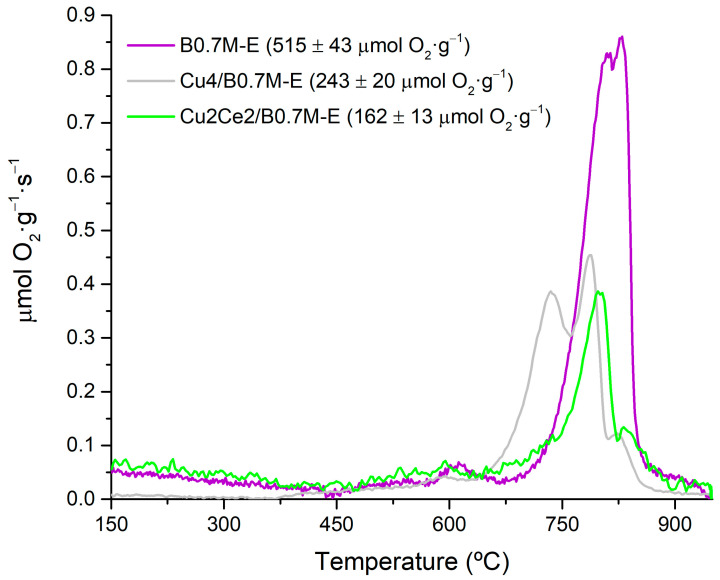
O_2_-TPD profiles of the B0.7M-E, Cu4/B0.7M-E and Cu2Ce2/B0.7M-E samples.

Figure 10 displays the O_2_ emission profiles during the O_2_-TPD experiments for the three samples under comparison. For perovskites, these profiles typically include the following signals: (i) between 150 and 350 °C, a peak that corresponds to the release of oxygen adsorbed on the surface defects (called α-O_2_); (ii) between 350 and 700 °C, the peak due to the desorption of oxygen from the lattice defects (α’-O_2_); and (iii) above 700 °C, the peak corresponding to the release of lattice oxygen (β-O_2_), which is linked with the reduction of Mn (IV) and Cu (II) according to the Reactions (9) and (10) [47,103,104]. Note that, as the CeO_2_ phase does not release oxygen under the tested conditions, the contribution due to the Ce(IV)/Ce(III) redox pair should not be observed [105].

2 Mn(IV) + O* ⇆ 2 Mn(III) + *V_O_* + 1/2 O_2_ (9)

2 Cu(II) + O* ⇆ 2 Cu(I) + *V_O_* + 1/2 O_2_ (10)

In reactions (9) and (10), O* represents an oxygen atom located in an oxygen site, and *V_O_* means an oxygen vacancy. Considering this information, the lower amount of oxygen detected for Cu2Ce2/B0.7M-E could be due not only to the coverage of the active sites by the impregnated phases, but also to the presence of a lower proportion of Mn (IV) in the bulk of the perovskite, as deduced for the surface from the XPS data. Additionally, note that the oxygen emission that comes from the CuO phase at around 700 °C [34] is not detected in the presence of cerium, which will also contribute to the decrease of the total oxygen emission. On the other hand, according to the proposal of X. Tan and coworkers [106], the formation of oxygen defects in the Cu-Ce solid solution promotes an oxygen emission at temperatures below 150 °C. For the Cu2Ce2/B0.7M-E sample this emission is not detected, as these oxygen species should be released during the pretreatment of the sample and, consequently, they have not been registered during the O_2_-TPD.

#### 3.4.4. Catalytic Activity

**Figure 11:**
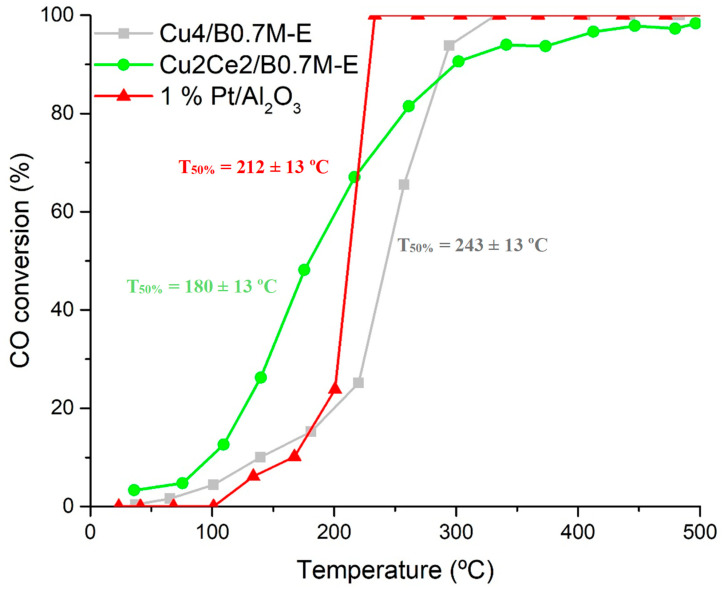
CO conversion profiles of the Cu4/B0.7M-E, Cu2Ce2/B0.7M-E and 1% Pt/Al_2_O_3_ samples under the 1% CO, 1% O_2_ in He reactant mixture.

The Cu2Ce2/B0.7M-E sample was tested for the CO oxidation reaction under CO-TPRe conditions, being the CO conversion profiles under the 1% CO, 1% O_2_ in He reactant atmosphere compared with those of Cu4/B0.7M-E and of 1% Pt/Al_2_O_3_ (used as reference) samples in Figure 11. The bimetallic sample exhibits a higher CO conversion than the monometallic one at temperatures between 100 and 200 °C, being also higher than the shown by the Pt-based sample, being consistent with published data for copper-ceria formulations [107,108].

**Figure 12:**
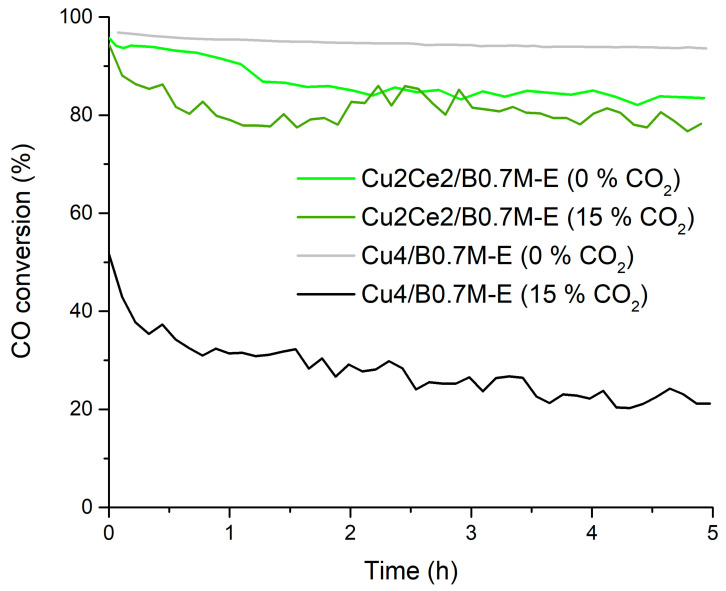
CO conversion profiles at 300 °C of the Cu4/B0.7M-E and Cu2Ce2/B0.7M-E samples in the absence and in the presence of 15% CO_2_.

The Cu2Ce2/B0.7M-E formulation was also tested at 300 °C under the 1% CO, 1% O_2_ in He reactant atmosphere, both, in the absence and in the presence of 15% CO_2_. According to the results presented in Figure 12 and in Table 8, the bimetallic sample shows an interesting performance under the two reactant atmospheres, as it displays a notably higher CO specific activity and a significantly lower deactivation by CO_2_ than the Cu4/B0.7M-E sample. Thus, the carbonation degree of the active sites should be lower in the presence of Ce, as the CeO_2_ phase should cover the Ba sites susceptible to be carbonated, and, condequently, more active sites exist on the Cu-Ce sample. Other authors also proposed that the improvement of the performance for CO oxidation in the presence of CO_2_ of a Cu-Ce system, despite being the CeO_2_ phase susceptible to carbonation (due to its high basicity [109,110]), is due to the interaction between CeO_2_ and CuO that allows overcoming this drawback [111,112].

**Figure 13:**
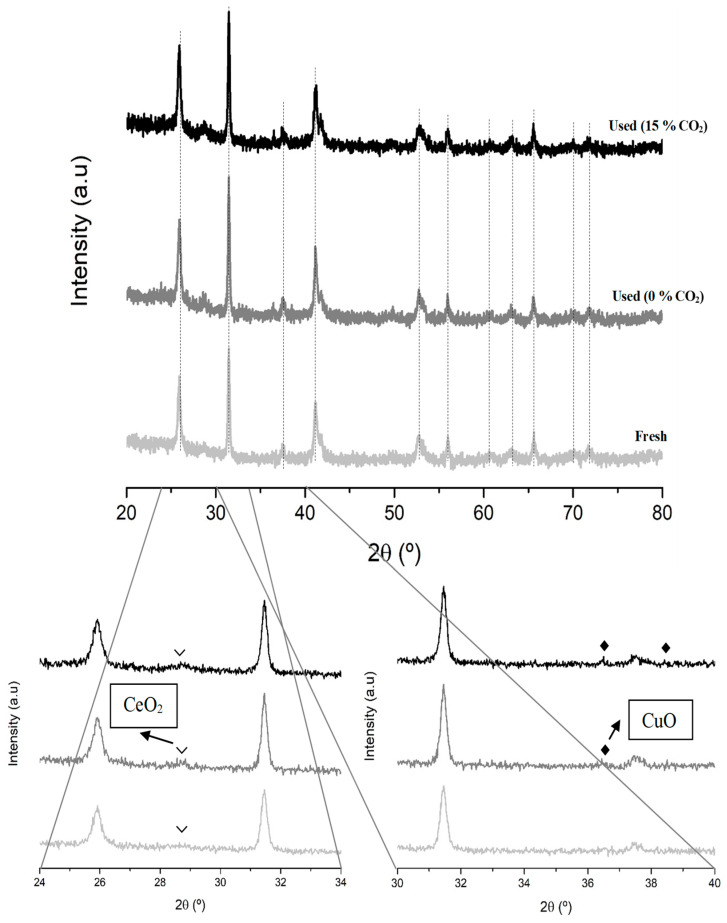
XRD profiles of the fresh and used Cu2Ce2/B0.7M-E sample in the CO oxidation tests, under the 1% CO, 1% O_2_ in He atmosphere, in the absence and in the presence of 15% CO_2_.

The XRD and XPS data of the Cu2Ce2/B0.7M-E sample used in the CO oxidation tests under both reactant atmosphere (Figure 13 and Table 9, respectively) provide interesting insights. The characterization data for the Cu4/B0.7M-E used sample have already been presented and discussed in the first section of this paper. Firstly, the XRD profiles indicate that the perovskite structure is not modified during the reaction, as it is also identified for the used sample. However, the XRD signals corresponding to the CuO and CeO_2_ phases are more intense in the used samples than in the fresh ones. On the other hand, the lower Mn(IV)/Mn(III) ratios of the used samples reveal a decrease of the Mn (IV) amount, and the significant decrease of the Ce/(Ba+Mn+Cu+Ce) ratio suggests that the amount of Ce species on the surface is lower after the reaction under the CO_2_-containing atmosphere. These two pieces of data seem to explain the decrease of the CO conversion over time. On the contrary, the amount of oxygen defects and of carbonate groups remained unchanged, indicating the high tolerance of the Cu2Ce2/B0.7M-E formulation to CO_2_. The analysis of Auger Cu L_3_M_4.5_M_4.5_ signal for the used samples reveals a clear shift towards lower kinetic energies. However, the similar percentages of the different Cu species on the surface reveals the high stability of Cu2Ce2/B0.7M-E under both conditions.

Finally, in order to test the stability of the Cu2Ce2/B0.7M-E sample at a longer reaction time, a stability test at 300 °C during 20 h and under the CO_2_-containing atmosphere was developed. As a low decrease of the CO conversion is registered (see Figure S5 in the Supplementary Information File) along the reaction time (ΔC_CO_ = −5%), it seems that the effect of cerium would be maintained during long periods of time.

## 4. Conclusions

In this paper, the Ba_0.8_Mn_0.7_Cu_0.3_O_3_ (B0.8MC) and the Cu(4 wt%)/Ba_0.7_MnO_3_ (Cu4/B0.7M-E) samples, selected from previous studies developed by the authors [33,34], were subjected to CO oxidation catalytic tests under more realistic conditions, i.e., in the presence of CO_2_ in the reactant atmosphere. Additionally, in order to improve the performance of the Cu4/B0.7M-E sample, the Cu(2 wt%)Ce(2 wt%)/Ba_0.7_MnO_3_ (Cu2Ce2/B0.7M-E) bimetallic formulation was synthesized, characterized and tested. According to the results presented and discussed, the following conclusions can be drawn:

(i) The B0.8MC sample showed the best catalytic performance during an isothermal reaction at 250 °C, but it featured a lower tolerance to CO_2_ than the Cu4/B0.7M-E sample, which was selected to be optimized by the addition of cerium.
(ii) The impregnation of the B0.7M-E support with cerium and copper did not cause significant structural changes in the perovskite structure.
(iii) The presence of cerium increased the fraction of the reduced copper species on the surface, improving the redox properties of the raw B0.7M-E support due to Mn-Ce synergistic effect.
(iv) The Cu2Ce2/B0.7M-E bimetallic formulation exhibits a better catalytic performance for the CO oxidation reaction than the monometallic one, including a notably higher tolerance to CO_2_.

## Figures and Tables

**Table 1:** Characterization and specific activity for CO oxidation (300 °C, under a 1% CO, 1% O_2_ in He reactant mixture) of the B0.8MC, Cu4/B0.7M-E and Cu12/B0.8M-E samples.

**Sample**	**XPS ^1^**					
	**Mn(IV)/Mn(III) ^2^**	**Cu/(Ba+Mn+Cu)** **(Nominal)**		**Cu_si_/Cu_wi_**		**O_L_/(Ba+Mn(+Cu))** **(Nominal) ^3^**
B0.8MC	0.96 ± 0.10	0.08 ± 0.01 (0.17)		1.40 ± 0.09		0.86 ± 0.03 (1.67)
Cu4/B0.7M-E	1.28 ± 0.05	0.10 ± 0.01 (0.07)		1.62 ± 0.07		1.41 ± 0.02 (1.76)
Cu12/B0.7M-E	1.34 ± 0.10	0.18 ± 0.01 (0.15)		1.30 ± 0.04		1.15 ± 0.03 (1.76)
**Sample**	**H_2_-TPR**		**O_2_-TPD**		**CO Specific Activity ^4^**	
	**mL H_2_ ·(g sample)^−1^**		**μ** **mol O_2_ ·(g sample)^−1^**			
B0.8MC	60 ± 15		367 ± 35		0.424 ± 0.048/0.401 ± 0.048	
Cu4/B0.7M-E	70 ± 15		243 ± 20		1.233 ± 0.043/1.231 ± 0.043	
Cu12/B0.7M-E	90 ± 16		100 ± 10		0.588 ± 0.042/0.588 ± 0.042	

^1^ The experimental ratios obtained by XPS were calculated by using the area under the deconvoluted signals, while the nominal values (shown between parentheses) are determined using the molecular formula of the samples. ^2^ Obtained through the analysis of the Mn 2p^3/2^ transition. For those calculated by the Mn 2p^1/2^ transition, see Table S1 and Figure S1 (in Supplementary Information File). ^3^ The contribution of the Cu 2p signal to the ratio is only applied for the B0.8MC sample. ^4^ As mol of CO converted per mol of Cu and per minute (first cycle/second cycle).

**Table 2:** CO conversion (ΔC_CO_) and CO specific activity (a_CO_) during the CO oxidation reaction at 250 °C for the B0.8MC and Cu4/B0.7M-E samples.

Sample	B0.8MC		Cu4/B0.7M-E	
Cycle	1	2	1	2
ΔC_CO_ (%) ^1^	−10 ± 6	−8 ± 6	−26 ± 6	−15 ± 6
a_CO_ (5 h) ^2^	0.309 ± 0.062	0.281 ± 0.067	0.518 ± 0.087	0.469 ± 0.094

^1^ Calculated as the difference between the final (5 h) and the initial CO conversions. ^2^ As mol of CO converted per mol of surface Cu and per minute.

**Table 3:** Ba, Mn, O and Cu XPS characterization data of the fresh and used B0.8MC and Cu4/B0.7M-E in the stability tests performed at 250 °C.

**Sample**	**Mn(IV)/Mn(III) ^1^**	**Cu/(Ba+Mn+Cu)**		**O_L_/(Ba+Mn(+Cu)) ^2^**		**BaCO_3_/Ba_L_**
Fresh B0.8MC	0.96 ± 0.10	0.08 ± 0.01		0.86 ± 0.03		0.15 ± 0.01
Used B0.8MC	0.58 ± 0.01	0.09 ± 0.01		0.18 ± 0.01		0.15 ± 0.01
Fresh Cu4/B0.7M-E	1.28 ± 0.05	0.10 ± 0.01		1.41 ± 0.02		0.16 ± 0.01
Used Cu4/B0.7M-E	0.79 ± 0.01	0.10 ± 0.01		0.22 ± 0.01		0.11 ± 0.01
**Sample**	**KE Cu L_3_M_4.5_M_4.5_ (eV) ^3^**		**Cu (II) (%) ^4^**		**Cu_si_ (%) ^4,5^**	
Fresh B0.8MC	917.5 ± 0.1		22 ± 12		78 ± 18	
Used B0.8MC	917.2 ± 0.1		42 ± 2		58 ± 5	
Fresh Cu4/B0.7M-E	918.8 ± 0.1		55 ± 5		45 ± 14	
Used Cu4/B0.7M-E	917.5 ± 0.1		21 ± 4		79 ± 5	

^1^ Obtained through the analysis of the Mn 2p^3/2^ transition. For those calculated by the Mn 2p^1/2^ transition, see Table S1 and Figure S1 (in Supplementary Information File). ^2^ The contribution of the Cu 2p signal to the ratio is only applied for the B0.8MC sample, since the lattice oxygen is mainly present in the perovskite support, and the CuO impregnated phase of Cu4/B0.7M-E is treated as an independent species. ^3^ KE = Kinetic energy. ^4^ Obtained by using the Cu L_3_M_4.5_M_4.5_ Auger signal. ^5^ Cu species with a higher interaction with the perovskite.

**Table 4:** Evolution of the CO conversion (ΔC_CO_) and CO specific activity (a_CO_) during the CO oxidation reaction at 300 °C (under a 1% CO, 1% O_2_ in He reactant atmosphere) in the presence of 15% CO_2_ for the B0.8MC and Cu4/B0.7M-E samples.

Sample	B0.8MC	Cu4/B0.7M-E
ΔC_CO_ (%) ^1^	- ^3^	−31 ± 6
a_CO_ (5 h) ^2^	0.012 ± 0.001	0.457 ± 0.030

^1^ Calculated as the difference between the final (5 h) and the initial CO conversions. ^2^ As mol of CO converted per mol of surface Cu and per minute. ^3^ Not applied for B0.8MC since it resulted to be inactive under the tested conditions.

**Table 5:** Ba, Mn, O and Cu XPS characterization data of the fresh and used B0.8MC and Cu4/B0.7M-E in the stability tests performed at 300 °C in the presence of 15% CO_2_.

**Sample**	**Mn(IV)/Mn(III) ^1^**		**Cu/(Ba+Mn+Cu)**		**O_L_/(Ba+Mn(+Cu)) ^2^**		**BaCO_3_/Ba_L_**
Fresh B0.8MC	0.96 ± 0.01		0.08 ± 0.01		0.86 ± 0.03		0.15 ± 0.01
Used B0.8MC	0.84 ± 0.03		0.04 ± 0.01		0.17 ± 0.01		0.06 ± 0.01
Fresh Cu4/B0.7M-E	1.28 ± 0.05		0.10 ± 0.01		1.41 ± 0.02		0.16 ± 0.01
Used Cu4/B0.7M-E	0.45 ± 0.02		0.05 ± 0.01		0.20 ± 0.01		0.41 ± 0.01
**Sample**		**KE Cu L_3_M_4.5_M_4.5_ (eV) ^3^**		**Cu (II) (%) ^4^**		**Cu_si_ (%) ^4,5^**	
Fresh B0.8MC		917.5 ± 0.1		22 ± 12		78 ± 18	
Used B0.8MC		917.6 ± 0.1		37 ± 8		63 ± 16	
Fresh Cu4/B0.7M-E		918.8 ± 0.1		55 ± 5		45 ± 14	
Used Cu4/B0.7M-E		917.6 ± 0.1		61 ± 4		39 ± 19	

^1^ Obtained through the analysis of the Mn 2p^3/2^ transition. See Table S1 and Figure S1 for those calculated by the Mn 2p^1/2^ transition. ^2^ The contribution of the Cu 2p signal to the ratio is only applied for the B0.8MC sample, since the lattice oxygen is mainly present in the perovskite support, and the CuO impregnated phase of Cu4/B0.7M-E is treated as an independent species. ^3^ KE = Kinetic energy. ^4^ Obtained by using the Cu L_3_M_4.5_M_4.5_ Auger signal. ^5^ Cu species with a higher interaction with the perovskite.

**Table 6:** XRD data of the fresh B0.7M-E, Cu4/B0.7M-E and Cu2Ce2/B0.7M-E samples.

Sample	Cell Parameters ^1^		Perovskite Average Crystal Size (nm)	Lattice Strain
	a (Å)	c (Å)		
B0.7M-E	5.68 ± 0.21	4.82 ± 0.32	26.2 ± 2.1	0.0002 ± ^2^
Cu4/B0.7M-E	5.69 ± 0.20	4.80 ± 0.28	15.2 ± 2.3	0.0017 ± 0.0004
Cu2Ce2/B0.7M-E	5.68 ± 0.20	4.79 ± 0.27	32.3 ± 2.8	0.0030 ± 0.0003

^1^ As the relationship between the cell parameters in the hexagonal crystal system is *a* = *b* ≠ c, *b* parameter is not shown in the table. ^2^ 6·10^−5^.

**Table 7:** Ba, Mn, O and Cu and Ce XPS characterization data of the fresh B0.7M-E, Cu4/B0.7M-E and Cu2Ce2/B0.7M-E samples.

**Sample**	**BaCO_3_/Ba_L_**	**Mn(IV)/Mn(III) ^1^**		**Mn(III)_c_/Mn(III)_f_**		**O_L_/(Ba+Mn)** **(Nominal = 1.76)**
B0.7M-E	0.08 ± 0.01	0.48 ± 0.01		-		1.06 ± 0.01
Cu4/B0.7M-E	0.16 ± 0.01	1.28 ± 0.05		0.18 ± 0.03		1.41 ± 0.02
Cu2Ce2/B0.7M-E	0.10 ± 0.01	1.05 ± 0.03		0.31 ± 0.02		0.29 ± 0.01
**Sample**	**Cu/(Ba+Mn+Cu(+Ce))** **(nominal)**		**Ce(IV)/Ce(III)**		**Ce/(Ba+Mn+Cu+Ce)** **(nominal)**	
B0.7M-E	-		-		-	
Cu4/B0.7M-E	0.10 ± 0.01 (0.07)		-		-	
Cu2Ce2/B0.7M-E	0.07 ± 0.01 (0.04)		1.83 ± 0.06		0.47 ± 0.01 (0.02)	
**Sample**	**Cu (II) (%) ^2^**			**Cu_red_/Cu_si_ (%) ^2,3^**		
B0.7M-E	-			-		
Cu4/B0.7M-E	55 ± 5			45 ± 14		
Cu2Ce2/B0.7M-E	41 ± 5			59 ± 16		

^1^ Obtained through the analysis of the Mn 2p^3/2^ transition. See Table S1 and Figure S1 for those calculated by the Mn 2p^1/2^ transition. ^2^ Obtained by using the Cu L_3_M_4.5_M_4.5_ Auger signal. ^3^ For Cu4/B0.7M-E, the Cu_si_ parameter is still being used (see Table 3 and Table 5), meanwhile for Cu2Ce2/B0.7M-E, Cu_red_ parameter is referred to Cu species with an oxidation state lower than Cu (II).

**Table 8:** Evolution of the CO conversion (ΔC_CO_) and CO specific activity (a_CO_) during the CO oxidation reaction at 300 °C in the absence and in the presence of 15% CO_2_ for the B0.8MC and Cu4/B0.7M-E samples.

Sample	Cu2Ce2/B0.7M-E		Cu4/B0.7M-E	
CO_2_ Content (%)	0	15	0	15
ΔC_CO_ (%) ^1^	−13 ± 6	−20 ± 6	−2 ± 6 ^3^	−31 ± 6
a_CO_ (5 h) ^2^	2.274 ± 0.108	1.620 ± 0.097	1.231 ± 0.043	0.457 ± 0.030

^1^ Calculated as the difference between the final (5 h) and the initial CO conversions. ^2^ As mol of CO converted per mol of surface Cu and per minute. ^3^ The high error is due to the association of an absolute error of the CO conversions of 3%, which was determined through a repeatability procedure. Despite this observation, the presented result is consistent with the previous study performed by the authors [34].

**Table 9:** Ba, Mn, O and Cu and Ce XPS characterization data of the fresh and used Cu2Ce2/B0.7M-E sample (CO oxidation tests, under the 1% CO, 1% O_2_ in He atmosphere, in the absence and in the presence of 15% CO_2_).

**Sample**	**BaCO_3_/Ba_L_**	**Mn(IV)/Mn(III) ^1^**	**Mn(III)_c_/Mn(III)_f_**	**O_L_/(Ba+Mn)** **(Nominal = 1.76)**
Fresh	0.10 ± 0.01	1.05 ± 0.03	0.31 ± 0.01	0.29 ± 0.01
Used (0% CO_2_)	0.09 ± 0.01	0.75 ± 0.01	0.31 ± 0.01	0.29 ± 0.01
Used (15% CO_2_)	0.08 ± 0.01	0.77 ± 0.02	0.35 ± 0.02	0.29 ± 0.01
**Sample**	**Cu/(Ba+Mn+Cu+Ce)** **(Nominal = 0.04)**	**KE Cu** **L_3_M_4.5_M_4.5_ (eV) ^2^**	**Ce(IV)/Ce(III)**	**Ce/(Ba+Mn+Cu+Ce)** **(Nominal = 0.02)**
Fresh	0.07 ± 0.01	918.2 ± 0.1	1.83 ± 0.06	0.47 ± 0.01
Used (0% CO_2_)	0.07 ± 0.01	917.9 ± 0.1	2.37 ± 0.04	0.46 ± 0.01
Used (15% CO_2_)	0.06 ± 0.01	917.4 ± 0.1	2.57 ± 0.06	0.44 ± 0.01
**Sample**	**Cu (II) (%) ^2^**		**Cu_red_ (%) ^3,4^**	
Fresh	41 ± 5		59 ± 16	
Used (0% CO_2_)	42 ± 5		58 ± 14	
Used (15% CO_2_)	41 ± 6		59 ± 18	

^1^ Obtained through the analysis of the Mn 2p^3/2^ transition. See Table S1 and Figure S1 for those calculated by the Mn 2p^1/2^ transition. ^2^ KE = Kinetic energy. ^3^ Obtained by using the Cu L_3_M_4.5_M_4.5_ Auger signal. ^4^ Referred to Cu species with a lower oxidation state than Cu (II).

## Data Availability

Data will be made available on request.

## Supplementary Materials

The following supporting information can be downloaded at: https://www.mdpi.com/article/10.3390/nano15191467/s1, Table S1: Mn(IV)/Mn(III) ratios obtained by using the Mn 2p3/2 and Mn 2p1/2 transitions; Table S2: Data for the deconvolution of Cu, Cu_2_O and CuO references; Figure S1 (part 1): Deconvoluted Mn 2p1/2 profiles; Figure S1 (part 2): Deconvoluted Mn 2p1/2 profiles; Figure S2 (part 1): Deconvoluted Cu L3M4.5M4.5 profiles; Figure S2 (part 2): Deconvoluted Cu L3M4.5M4.5 profiles; Figure S3: EDX mapping analysis (Ba, Mn and Cu) of the Cu4/B0.7M-E and Cu2Ce2/B0.7M-E samples; Figure S4: EDX mapping analysis (Ce) of the Cu2Ce2/B0.7M-E sample; Figure S5: CO conversion profile at 300ºC of the Cu2Ce2/B0.7M-E sample in the presence of 15% CO_2_ during 20 h.

## Abbreviations

The following abbreviations are used in this manuscript:

GHGs	Greenhouse Gases
ICEs	Internal Combustion Engines
HCs	Hydrocarbons
ICP-OES	Inductively Coupled Plasma Optical Emission Spectroscopy
XPS	X-ray Photoelectron Spectroscopy
H_2_-TPR	Temperature-Programmed Reduction with H_2_
XRD	X-Ray Diffraction
JCPDS	Joint Committee on Powder Diffraction Standards
ICDD	International Centre for Diffraction Data
KE	Kinetic Energy
O_2_-TPD	Temperature-Programmed Desorption of O_2_
TPRe	Temperature-Programmed Reaction
TCD	Thermal Conductivity Detector
TG-MS	Thermo Gravimetric Mass Spectrometer
TWCs	Three-Way Catalysts
GHSV	Gas Hourly Space Velocity
FE-SEM	Field Emission Scanning Electron Microscopy
EDX	Energy Dispersive X-ray spectroscopy
