# Supplementary material for: An Approach to the Optimization of Ba-Mn-Cu Perovskites as Catalysts for CO Oxidation: The Role of Cerium
Source: Nanomaterials (Basel). 2025 Sep 25;15(19):1467. doi: 10.3390/nano15191467 (PMC12525801; doi:10.3390/nano15191467)
Supplement: Supplementary file 1 [file nanomaterials-15-01467-s001.zip › nanomaterials-3764701-supplementary.pdf]

An approach to the optimization of Ba-Mn-Cu perovskites as catalysts for CO oxidation: the role of cerium

Álvaro Díaz Verde, María José Illán Gómez

MCMA Group, Inorganic Chemistry Department, Materials Institute of the University of Alicante (IUMA), Faculty of Sciences, University of Alicante, 03690 Alicante, Spain

Table S1. Mn(IV)/Mn(III) ratios obtained by using the Mn $2p^{3/2}$ and Mn $2p^{1/2}$ transitions.

**Sample**	**Mn(IV)/Mn(III) (Mn $2p^{3/2}$)**	**Mn(IV)/Mn(III) (Mn $2p^{1/2}$)**
Fresh B0.8MC	0.96 ± 0.10	0.84 ± 0.06
Fresh Cu4/B0.7M-E	1.28 ± 0.05	1.61 ± 0.16
Fresh Cu12/B0.7M-E	1.34 ± 0.10	1.71 ± 0.33
Used B0.8MC (250ºC)	0.58 ± 0.01	0.50 ± 0.02
Used Cu4/B0.7M-E (250ºC)	0.79 ± 0.01	0.58 ± 0.02
Used B0.8MC (300ºC, 15 % CO_2)	0.84 ± 0.03	0.60 ± 0.04
Used Cu4/B0.7M-E (300ºC, 15 % CO_2)	0.45 ± 0.02	0.18 ± 0.01
Fresh B0.7M-E	0.48 ± 0.01	0.33 ± 0.02
Fresh Cu2Ce2/B0.7M-E	1.05 ± 0.03	1.06 ± 0.07
Used Cu2Ce2/B0.7M-E (300ºC, 0 % CO_2)	0.75 ± 0.01	0.66 ± 0.03
Used Cu2Ce2/B0.7M-E (300ºC, 15 % CO_2)	0.77 ± 0.02	0.66 ± 0.04

Table S1 displays the Mn(IV)/Mn(III) ratios obtained through the analysis of the Mn $2p^{3/2}$ and Mn $2p^{1/2}$ transitions. Note that some ratios are very similar, meanwhile others significantly differ, however, in spite of this finding, the trends of the Mn(IV)/Mn(III) ratios followed by both set of data are similar.

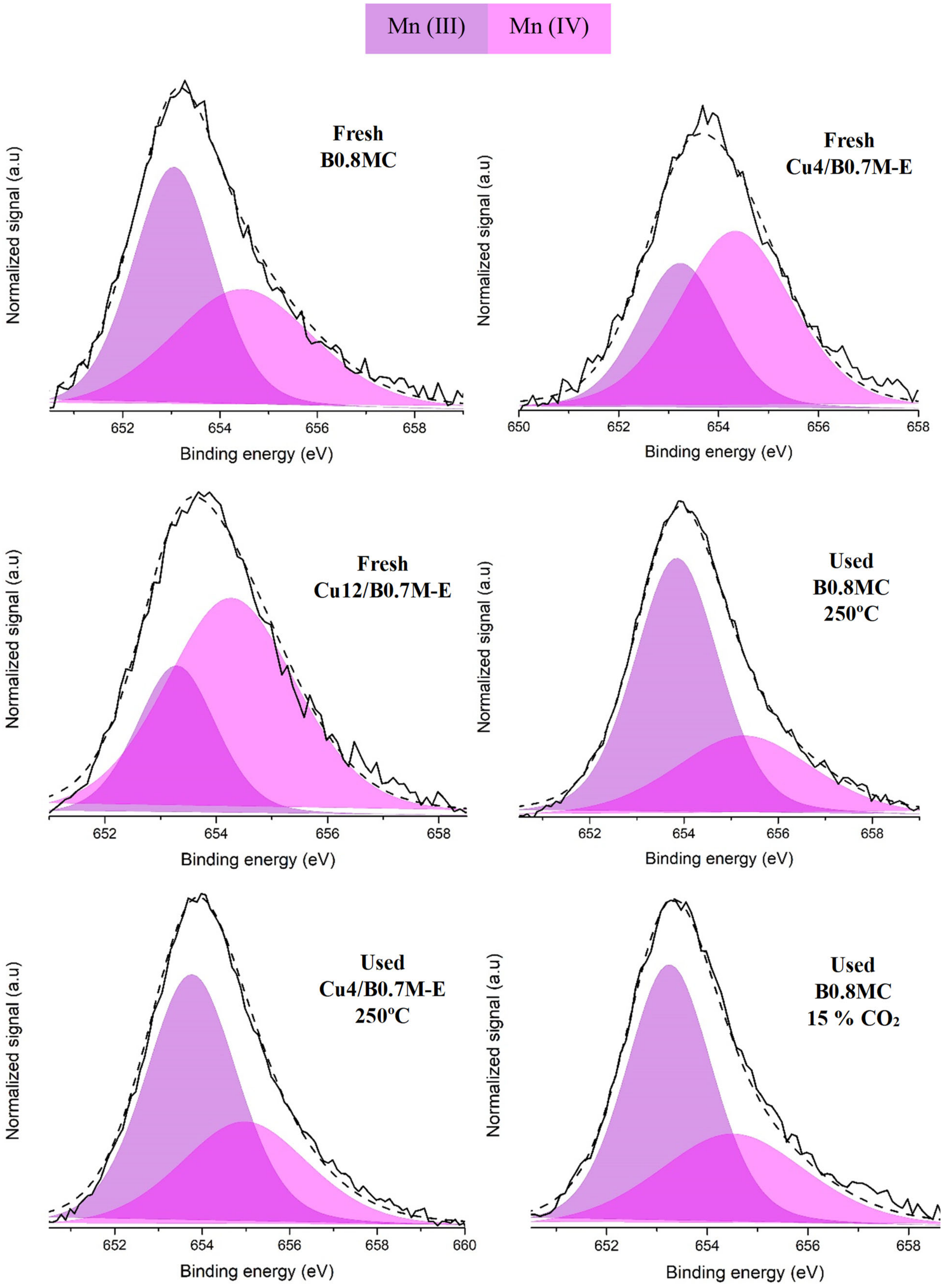

Figure S1 (part 1). Deconvoluted Mn $2p^{1/2}$ profiles.

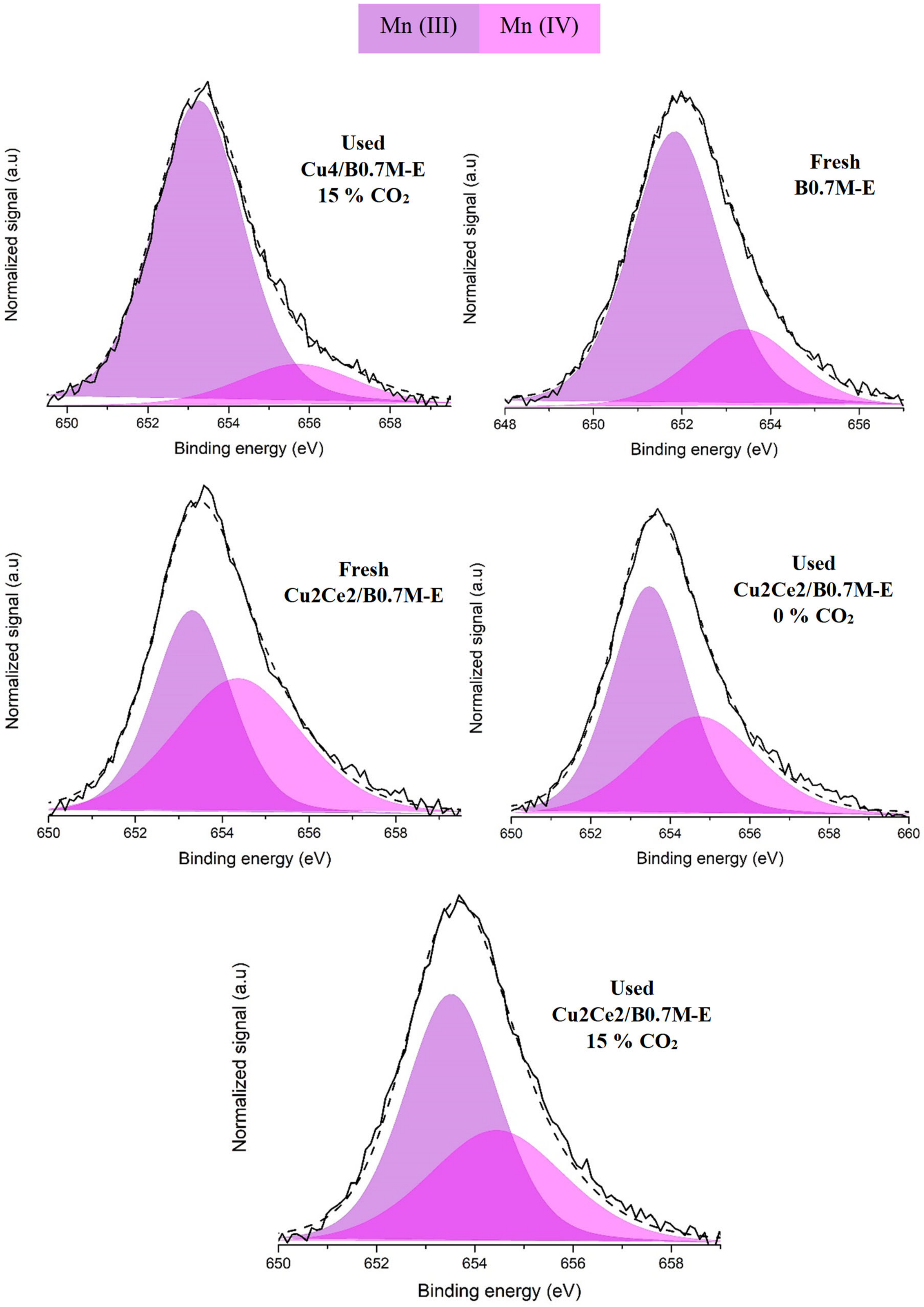

Figure S1 (part 2). Deconvoluted Mn $2p^{1/2}$ profiles.

Table S2. Data for the deconvolution of Cu, Cu_2O and CuO references.

	**Kinetic energy (eV)**	**FWHM (eV)**
**Cu**	911.16	3.50
	914.17	2.32
	916.25	1.54
	917.75	1.65
	918.66	0.95
	919.70	1.15
	921.38	1.50
**Cu_2O**	913.70	5.75
	917.00	2.10
	919.15	3.10
	922.10	2.35
**CuO**	911.50	2.85
	914.30	4.50
	917.90	2.20
	920.40	2.75

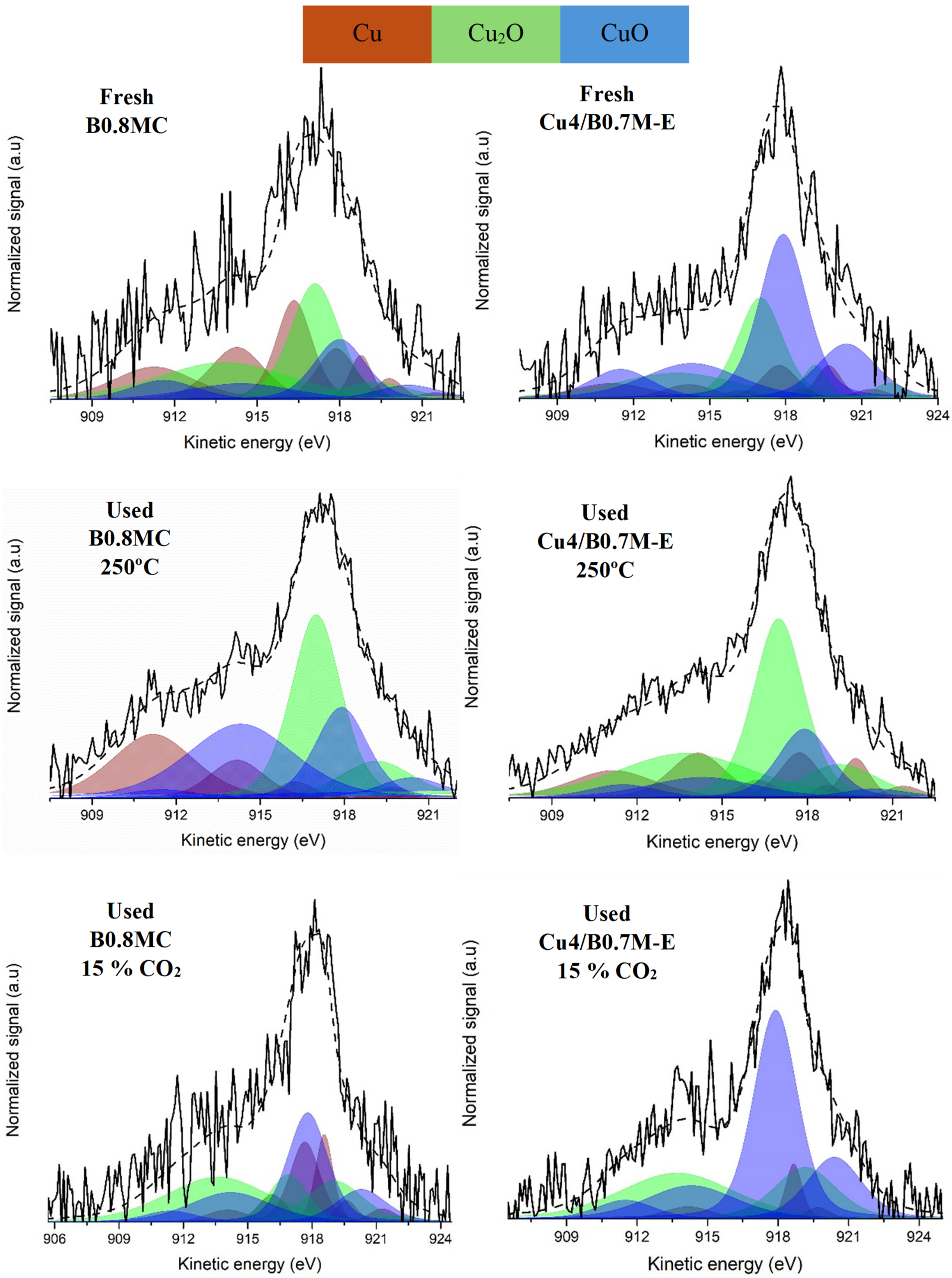

Figure S2 (part 1). Deconvoluted Cu $L_3M_{4.5}M_{4.5}$ profiles.

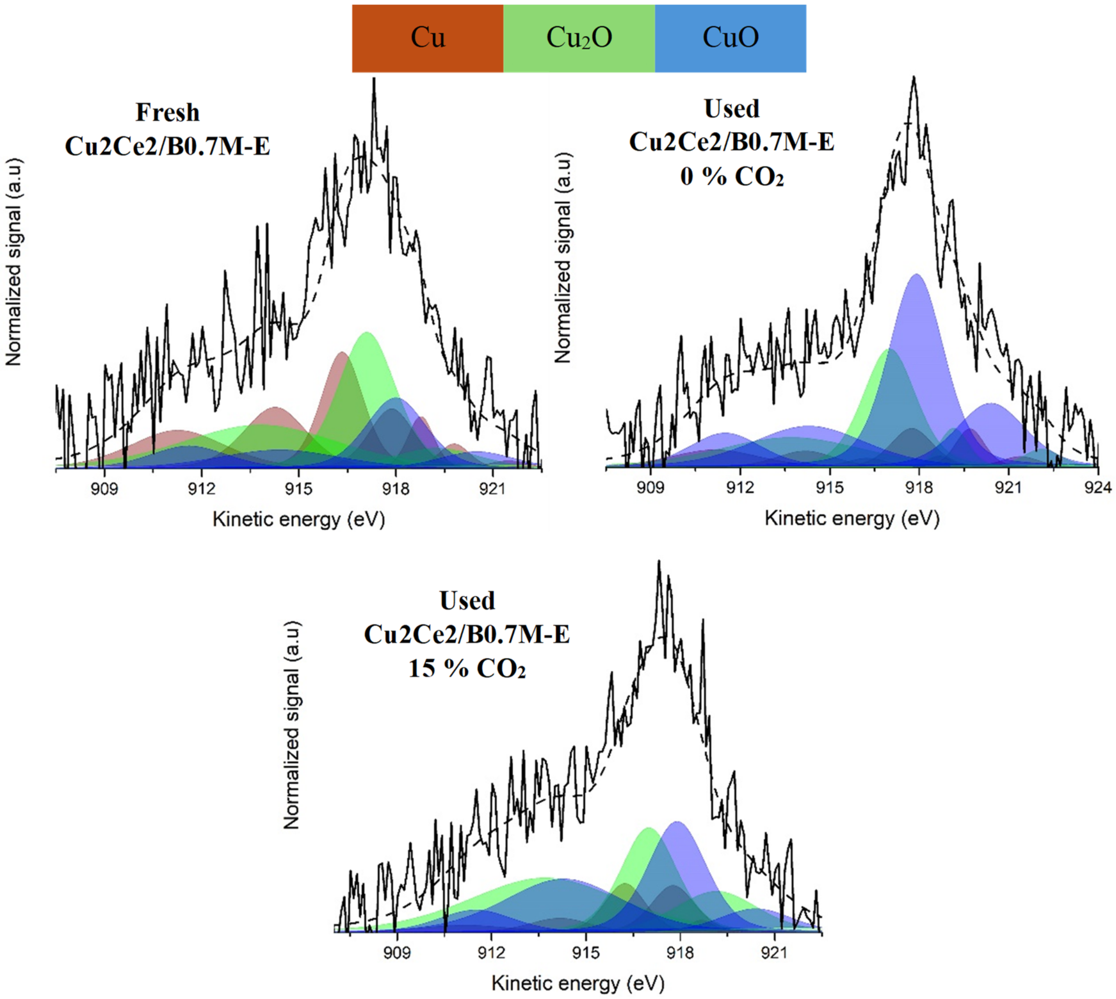

Figure S2 (part 2). Deconvoluted Cu $L_3M_{4.5}M_{4.5}$ profiles.

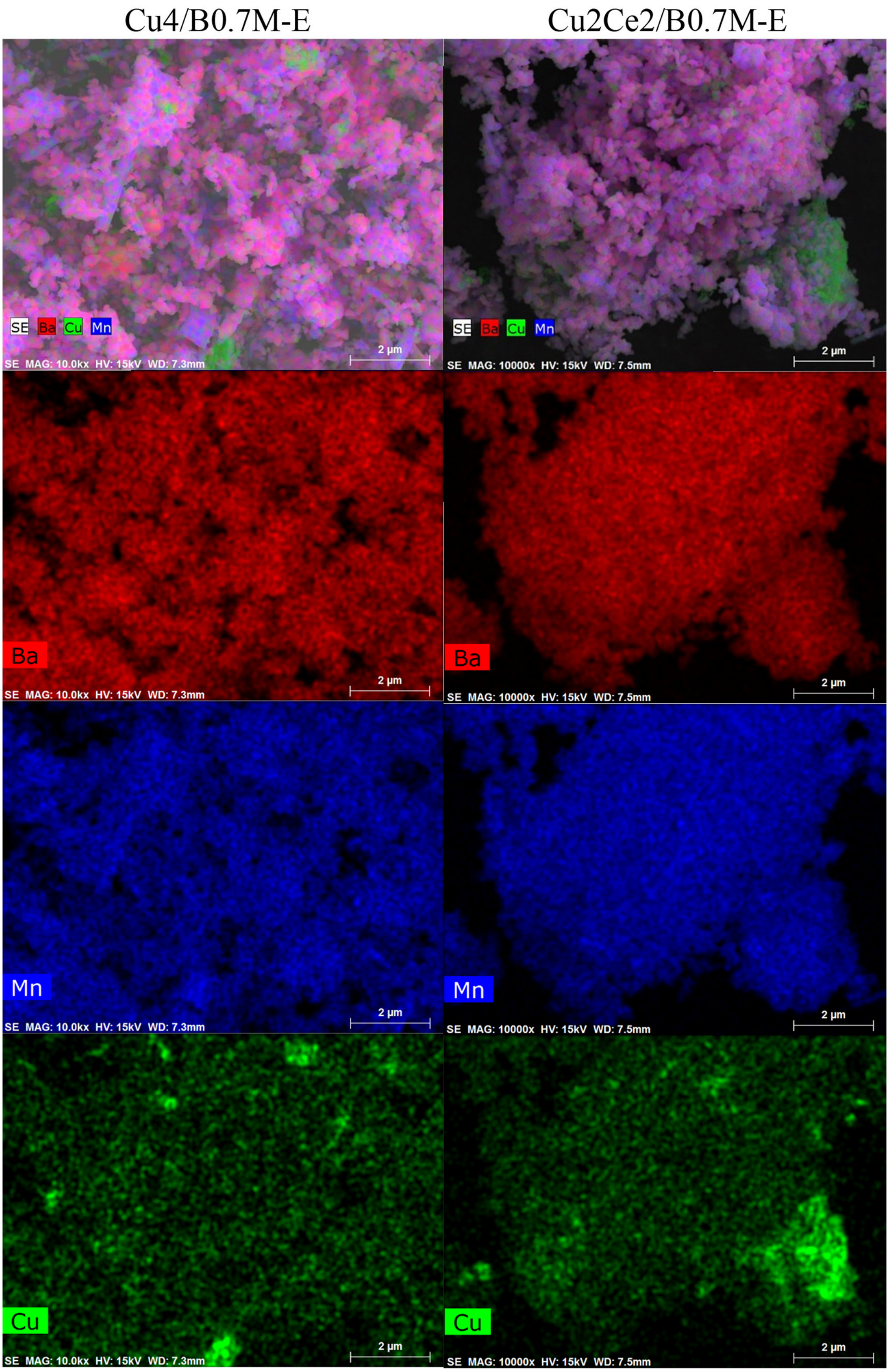

Figure S3. EDX mapping analysis (Ba, Mn and Cu) of the Cu4/B0.7M-E and Cu2Ce2/B0.7M-E samples.

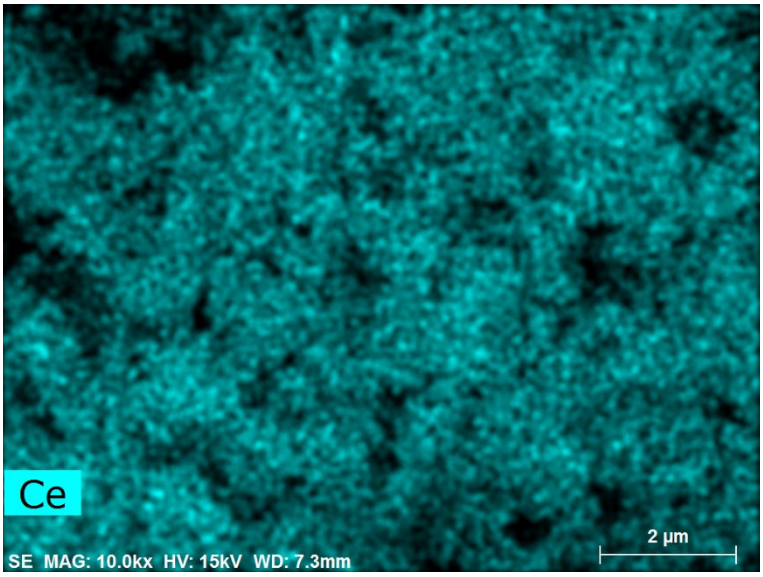

Figure S4. EDX mapping analysis (Ce) of the Cu2Ce2/B0.7M-E sample.

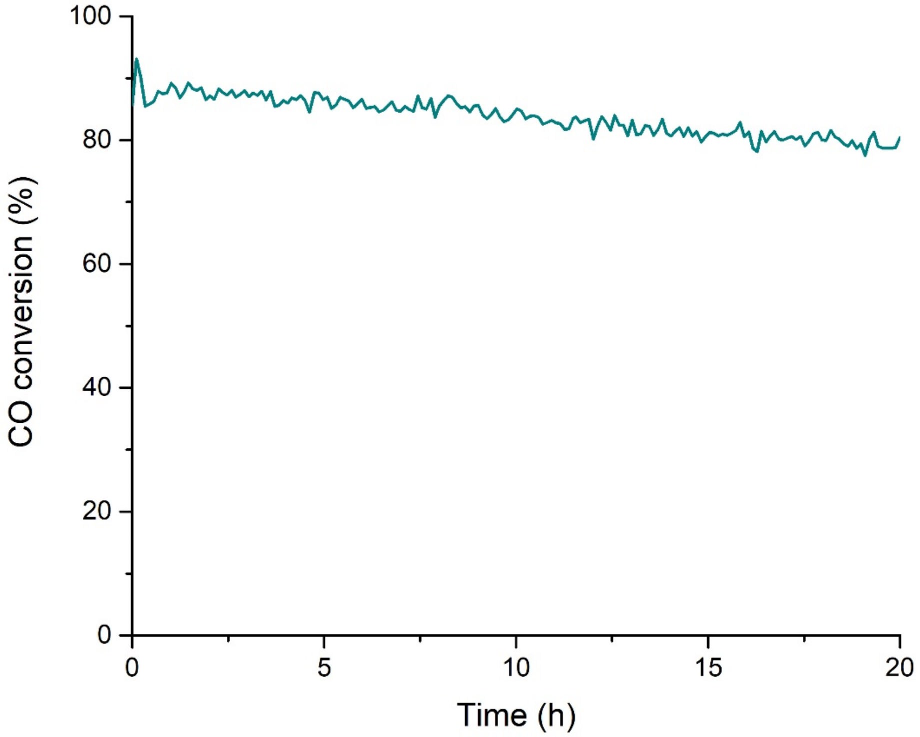

Figure S5. CO conversion profile at 300ºC of the Cu2Ce2/B0.7M-E sample in the presence of 15 % CO_2 during 20 h.
